# A Review of Current Trends with Type 2 Diabetes Epidemiology, Aetiology, Pathogenesis, Treatments and Future Perspectives

**DOI:** 10.2147/DMSO.S319895

**Published:** 2021-08-10

**Authors:** Josh Reed, Stephen Bain, Venkateswarlu Kanamarlapudi

**Affiliations:** 1 Institute of Life Science 1, Medical School, Swansea University, Swansea, SA2 8PP, UK

**Keywords:** diabesity, GLP-1, GLP-1R, incretin effect, insulin, metabolic homeostasis

## Abstract

Type 2 diabetes (T2D), which has currently become a global pandemic, is a metabolic disease largely characterised by impaired insulin secretion and action. Significant progress has been made in understanding T2D aetiology and pathogenesis, which is discussed in this review. Extrapancreatic pathology is also summarised, which demonstrates the highly multifactorial nature of T2D. Glucagon-like peptide (GLP)-1 is an incretin hormone responsible for augmenting insulin secretion from pancreatic beta-cells during the postprandial period. Given that native GLP-1 has a very short half-life, GLP-1 mimetics with a much longer half-life have been developed, which are currently an effective treatment option for T2D by enhancing insulin secretion in patients. Interestingly, there is continual emerging evidence that these therapies alleviate some of the post-diagnosis complications of T2D. Additionally, these therapies have been shown to induce weight loss in patients, suggesting they could be an alternative to bariatric surgery, a procedure associated with numerous complications. Current GLP-1-based therapies all act as orthosteric agonists for the GLP-1 receptor (GLP-1R). Interestingly, it has emerged that GLP-1R also has allosteric binding sites and agonists have been developed for these sites to test their therapeutic potential. Recent studies have also demonstrated the potential of bi- and tri-agonists, which target multiple hormonal receptors including GLP-1R, to more effectively treat T2D. Improved understanding of T2D aetiology/pathogenesis, coupled with the further elucidation of both GLP-1 activity/targets and GLP-1R mechanisms of activation via different agonists, will likely provide better insight into the therapeutic potential of GLP-1-based therapies to treat T2D.

## Introduction

Type 2 diabetes (T2D) is the most common and clinically important metabolic disorder which has become a global pandemic in recent decades and a major healthcare burden worldwide. In 2013, there were an estimated 382 million patients with diabetes globally.^1^ Concerningly, the T2D incidence continues to increase, and it is projected that there will be >590 million patients diagnosed with this condition by 2035.^1^,^2^ The World Health Organisation (WHO) defines diabetes as a “metabolic disorder of multiple aetiology characterised by chronic hyperglycaemia with disturbance of carbohydrate, fat, and protein metabolism resulting from defects in insulin secretion, insulin action, or both.”^3^ The most prevalent form of diabetes is type 2, as an estimated 90% of diabetes patients are diagnosed with this form,^2^ and the majority of the remaining 10% of patients have type 1 diabetes (T1D), although there are other rare types.^4^ T2D is largely caused by impaired insulin production and secretion by pancreatic beta-cells, as well as peripheral tissue insulin resistance.^5^,^6^ Given that ~90% of patients are obese or overweight at T2D diagnosis, the aetiology of T2D is largely thought to be linked to diets involving excessive nutrient consumption combined with insufficient energy expenditure.^7^ There are a range of effective treatments that reduce hyperglycaemia in T2D patients, which mediate their effects by improving insulin secretion or decreasing peripheral tissue insulin resistance.^8^ Despite this, post-diagnosis complications, especially long-term complications, are prevalent globally. As a result, diabetes remains a leading cause of blindness, end-stage renal disease, lower limb amputation and cardiovascular disease.^3^

T2D and obesity have such an interdependent relationship that the term “diabesity” has been coined.^9^ In recent decades, the number of people with T2D has more than doubled, and the increased global burden of T2D is thought to be largely due to an increase in obesity.^9^,^10^ Obesity has become a global pandemic over recent decades. Weight loss is associated with an improved prognosis for overweight T2D patients and obese individuals. Better glycaemic control has been reported in T2D patients who have lost weight, and excess body weight is associated with the risk of cardiometabolic complications, which are major causes of morbidity and mortality in T2D and obese individuals.^11^,^12^ Bariatric surgery has proven to be an effective treatment for diabesity, but it is expensive and there are numerous post-surgery complications: for example, vomiting and dumping syndrome, iron and B12 deficiency, and secondary hyperparathyroidism.^13^ GLP-1 analogues (eg liraglutide and exenatide) are used to treat T2D as they promote insulin secretion and induce weight loss.^3^ Since the GLP-1 receptor (GLP-1R) agonists are effective in treating diabesity, they could be pharmacological alternatives to bariatric surgery but without the post-surgery complications.^3^,^13^,^14^

The current literature on T2D epidemiology, aetiology, pathogenesis and treatment is discussed in this review.

## Obesity

**Figure 1:**
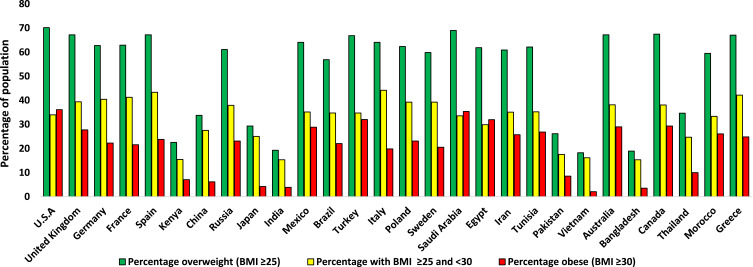
Variation in the percentage of obese and overweight individuals over 18 years of age in the population between different countries in 2016.^325^ Values displayed are an average of the male and female percentage of the population for each country, which were taken from the WHO data.^325^

Obesity is caused by excessive energy intake and subsequent storage coupled with insufficient energy expenditure resulting in weight gain. It has become a huge healthcare concern over the last few decades, especially in developed countries. However, in some obese individuals, excessive dietary intake may have a genetic aetiology, such as leptin deficiency.^15–17^ Obese individuals have a BMI of ≥30 kg/m^2^, and individuals with a BMI of ≥25 and <30 are classified as overweight.^16^ Obesity and overweight incidences have more than doubled since 1980, giving rise to >2.1 billion individuals with a BMI of >25 globally, as stated by the WHO.^15^,^18^ The WHO estimated that there were ~600 million obese individuals in 2014, and this number will continue to rise in the future.^18^ Obesity is associated with an increased risk for T2D, hypertension, dyslipidaemia, cardiovascular diseases, musculoskeletal disorders (such as osteoarthritis), certain types of cancer, and premature mortality.^19^,^20^ It is well established that there is a generally directly proportional relationship between BMI and both fasting and postprandial insulin levels.^21^,^22^ A similar relationship also exists between BMI and the degree of insulin resistance.^22^ The hyperinsulinemia associated with rising BMI is necessary to overcome insulin resistance and maintain normoglycemia.^22^ Not all obese individuals develop insulin resistance though;^23^ one study reported that 19, 34 and 60% of individuals with a BMI of <30, ≥30–35 and >35, respectively, were insulin resistant.^22^ Insulin hypersecretion also becomes more frequent with increasing BMI;^24^ 28, 49 and 80% of individuals with a BMI of <30, >30–35 and >35, respectively, exhibited insulin hypersecretion.^22^ Of the 2.1 billion individuals that are obese or overweight <25% have been diagnosed with T2D.^1^,^18^ The rise in T2D over the last few decades is generally thought to be attributed to an increase in the percentage of overweight individuals in the global population.^3^,^25^ Figure 1 shows how the percentage of overweight and obese individuals in the population vary between countries.

## Type 2 Diabetes

Diabetes mellitus was first reported over 3000 years ago in an Egyptian manuscript.^26^ However, it was not until 1936 that the distinction between T1D and T2D was made.^27^ T2D is by far the most common form of diabetes and accounts for ~90% of diabetes cases.^2^,^7^ T2D is a chronic metabolic complex multifactorial disease, involving many organs, that has become a global pandemic in recent decades.^1^,^28^ T2D is characterised by pancreatic beta-cell dysfunction, increased pancreatic alpha-cell function, and peripheral tissue insulin resistance. These alterations result in (1) hyperglycaemia due to impaired peripheral glucose uptake, (2) dyslipidaemia (hypertriglyceridemia and low high-density lipoprotein [HDL]-cholesterol) due to impaired peripheral fat uptake, (3) impaired amino acid uptake and ATP production into peripheral tissues, such as skeletal muscle, due to the impaired nutrient uptake, and (4) increased glucagon production, which further amplifies hyperglycaemia and hyperlipidaemia.^29–32^ T2D is thought to be largely provoked by diets involving excessive nutrient consumption, as ~90% of patients are obese or overweight in western countries.^3^,^9^,^16^,^33^ In contrast to that observed in western countries, a study in China found that 50.3% of T2D patients were not overweight,^34^ and other Asian countries also have a high percentage of T2D patients with a BMI of <25.^35^ It has become clear that genetic factors also play a role in conferring susceptibility, although no important risk genes have yet been identified.^36^,^37^ T2D is considered to be a heterogeneous disease as the severity of insulin deficiency, treatment requirements and prognosis varies between patients.^38^,^39^

Although treatments are available, diabetes remains a leading cause of blindness, end-stage renal disease, lower limb amputation and cardiovascular disease.^40^ One study in the UK found that 48.9% of amputations from 2007 to 2010 were carried out on people with diabetes and diabetes conferred a 23.3x risk for amputations.^41^ Additionally, diabetic patients also usually exhibit impaired wound healing, susceptibility to infection, neuropathology and impotence.^3^ T2D has also been identified as a risk factor for Alzheimer’s and Parkinson’s diseases.^42^,^43^ Cardiovascular disease (CVD) accounts for 75% of all related deaths in diabetic patients and on average individuals with T2D have an increased risk of shorter life expectancy.^44^,^45^ There are a range of treatments available which improve insulin secretion and/or decrease peripheral tissue insulin resistance, which reduces hyperglycaemia.^46^,^47^ However, maintaining glycaemic control in patients is no easy task, as hypo- or hyperglycaemia can result from administration of the currently available treatments resulting in either too much or not enough, respectively, of an anti-hyperglycaemic effect.^3^,^48^ Regardless of the treatment regime, longitudinal studies in humans have demonstrated that beta-cell function generally deteriorates over time.^49^,^50^ It has been postulated that chronic hyperglycaemia and dyslipidaemia lead to a progressive decline in beta-cell function post-diagnosis.^50^

### Epidemiology of T2D

It has been estimated that there were ~382 million T2D patients globally in 2013 and that number has more than doubled in the last few decades.^1^ The incidence and prevalence of T2D continue to increase. By 2035, it is estimated that there will be >590 million T2D people diagnosed.^1^,^2^ Although the prevalence and incidence of T2D vary between countries, T2D is still considered to be a global disease.^28^ T2D used to be considered as a disease induced by ‘western lifestyles’ (high-calorie diets and sedentary lifestyles).^3^ Interestingly, the rise in prevalence of T2D is estimated to be almost 4x as high in developing as in developed countries.^1^,^25^,^51^ This is thought to be due to developing countries adopting ‘western lifestyles’ and the increase in obesity and the number of people being overweight in their populations.^1^,^28^,^52^ In general, the age group with the highest risk of developing T2D is 40–60 years in developed countries and 60+ years in developing countries.^53^ Although T2D is considered to be a disease associated with adulthood and the incidence of T2D increases with age, it is becoming more common for children to be affected.^54^,^55^ It is, however, likely that the number of cases of individuals diagnosed with T1D in adulthood is underestimated: it has been speculated that 5–15% of adult patients are misdiagnosed as having T2D when they may actually have T1D, which is currently an area of controversy in the literature.^56^,^57^

**Figure 2:**
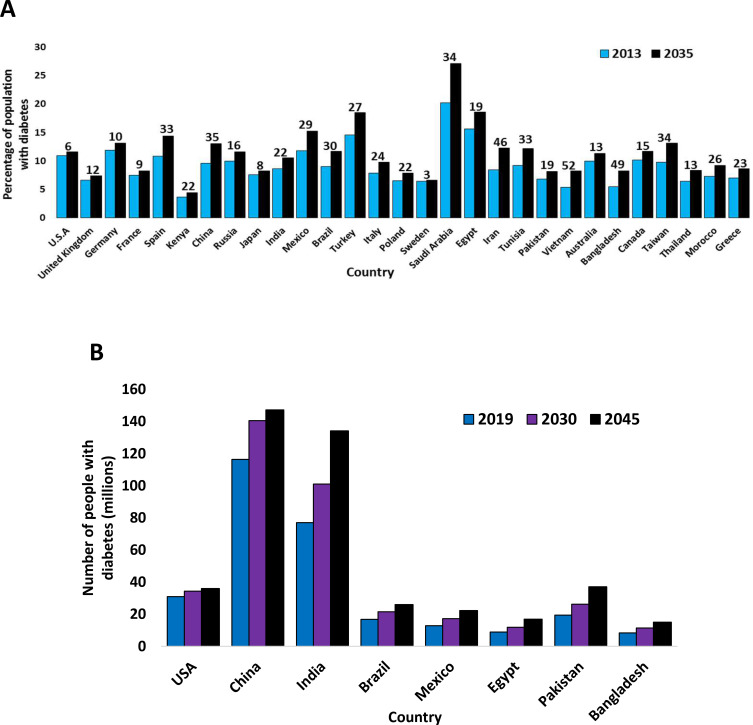
(**A**) The varying estimated prevalence of T2D in 2013 and projections for 2035, between ages 20–79 years.^1^ (**B**) The varying estimated prevalence of T2D in 2019 and projections for 2030 and 2045, between ages 20–79 years.^62^ The numbers above are the values for each country, indicating the percentage increase of diabetes patients from 2013 to 2035 (**A**) or from 2019 to 2045 (**B**) for any given country, rounded to the nearest whole number. Data from these studies.^1^,^62^

Diabetes in young people was previously thought to be T1D.^58^ Until the early 1990s, it was rare for paediatric centres to have T2D patients. However, this has drastically changed since the late 1990s onwards with paediatric T2D accounting for up to 45% of newly diagnosed paediatric diabetes cases in the USA. The ”youth T2D” is not just a phenomenon limited to the USA.^59^ Staggeringly, 80% of all new diabetes cases in Japan in children and adolescents were reported as T2D and other countries have also reported an increase in youth T2D.^60^ However, many studies from Europe have reported that youth T2D is much rarer, accounting for only 1–2% of all diabetes mellitus cases.^55^,^60^ China has the highest number of T2D patients (~98 million) and India has the second-highest (around 65 million).^1^ The USA was estimated to have around 24.4 million T2D patients in 2013.^61^ Tokelau has been reported in 2013 as the country with the greatest prevalence of T2D (37.5%) in the national population. The Federated States of Micronesia, the Marshall Islands, and Kiribati all have T2D prevalence rates in their national populations of 35, 34.9 and 28.8%, respectively.^1^ All other countries had T2D national population prevalence rates of under 26% in 2013.^1^ Globally, the number of people with diabetes was estimated to be 285, 366, 382, 415 and 425 million in the years 2009, 2011, 2013, 2015 and 2017, respectively.^62^ Figure 2 shows how T2D prevalence varied between countries in 2013 and 2019, and predictions for future data on T2D prevalence are also shown.^1^,^62^

Diabetes was considered to be rare in the first half of the twentieth century in the USA; less than 1% of the population was diagnosed with diabetes in 1958.^63^,^64^ However, both the incidence and prevalence of T2D increased throughout the second half of the 20th century in developed countries, becoming an epidemic towards the end of the century and remaining as such into the 21st century.^1^,^63^ Over recent decades, T2D incidence and prevalence have also increased in developing countries, becoming a comparable health burden in these countries. In 1980, <1% of China’s population had diabetes but this increased to almost 10% by 2008.^25^ In urban areas of South India, almost 20% of the population was estimated to be affected by diabetes in 2008.^65^ In 2014, a study revealed that 46% of newly diagnosed T2D patients in India were under 40 years of age,^66^ which is a contrast with the notion that patients in developing countries are usually over 60.^53^ T2D incidence and prevalence have continued to increase globally in the 21st century as well. By 2045, it has been estimated that there will be over 700 million T2D patients worldwide.^1^,^62^ However, as highlighted in Tables 1 and 2, the future increase in the number of T2D patients will be partly due to the rise in the global population and not just increasing incidence.^1^

**Table 1:** Variation of T2D Prevalence Between Global Regions in 2013 and Projections for 2035

Region	Population 2013 (20–79 Years) Millions	Diabetes Patients (20–79 Years) Millions	Comparative Diabetes Prevalence (20–79 Years) %	Population 2035 (20–79 Years) Millions	Diabetes Patients (20–79 Years) Millions	Comparative Diabetes Prevalence (20–79 Years) %
**Africa**	407.8	19.8	5.7	775.5	41.5	6
**Europe**	658.7	56.3	6.8	668.7	68.9	7.1
**North America and Caribbean**	334.9	36.7	9.6	404.5	50.4	12.3
**Middle-East and North Africa**	347.5	34.6	10.9	583.7	67.9	11.3
**South-East Asia**	883.2	72.1	8.7	1216.9	123	9.4
**South and Central America**	300.5	24.1	8.2	394.2	38.5	8.2
**Western Pacific**	1613.2	138.2	8.1	1818.2	201.8	8.4

**Notes:** Adapted from *Diabetes Res Clin Pract*, 103(2), Guariguata L, Whiting DR, Hambleton I, Beagley J, Linnenkamp U, Shaw JE. Global estimates of diabetes prevalence for 2013 and projections for 2035. *Diabetes Res Clin Pract*. 137–149. Copyright (2014), with permission from Elsevier.^1^

**Table 2:** Variation of T2D Prevalence Between Global Regions in 2019 and Projections for 2030 and 2045

Region	Diabetes Patients in 2019 (20–79 Years) Millions	Comparative Diabetes Prevalence (20–79 Years) %	Diabetes Patients in 2030 (20–79 Years) Millions	Comparative Diabetes Prevalence (20–79 Years) %	Diabetes Patients in 2045 (20–79 Years) Millions	Comparative Diabetes Prevalence (20–79 Years) %
**Africa**	19.4	4.7	28.6	5.1	47.1	5.2
**Europe**	59.3	6.3	66	7.3	68.1	7.8
**North America and Caribbean**	47.6	11.1	56	12.3	63.2	13
**Middle-East and North Africa**	54.8	12.2	76	13.3	107.6	13.9
**South-East Asia**	87.6	11.3	115.1	12.2	152.8	12.6
**South and Central America**	31.6	8.5	40.2	9.5	49.1	9.9
**Western Pacific**	162.2	11.4	196.5	12.4	212.2	12.8

**Notes:** Adapted from *Diabetes Res Clin Pract*, 2, Saeedi P, Petersohn I, Salpea P, et al. Global and regional diabetes prevalence estimates for 2019 and projections for 2030 and 2045: results from the International Diabetes Federation Diabetes Atlas, 9th edition. 157, copyright (2019), with permission from Elsevier.^62^

Studies have demonstrated that the incidence of T2D potently increased globally in the latter half of the 20^th^ century (especially paediatric cases), and one study even reported the incidence of diabetes rose from 2.6 to 9.4% between 1980 and 1988 in the USA.^59^,^67^ Although life expectancy has generally increased globally over the last few decades, this does not fully explain the rise in T2D incidence and prevalence given the drastic increase in childhood cases, but it likely accounts for at least part of the rise in T2D in developing countries where patients are generally aged 60+ years.^47^ Globally, it was estimated that diabetes accounted for ~12% of health expenditures in 2010 (at least $376 billion), and the healthcare cost will continue to rise to ~$490 billion in 2030.^28^ Many developing countries do not have adequate infrastructure to treat this pandemic, meaning that diabetes is a serious concern for the future.^28^ Additionally, there is continuing emerging evidence that the prevalence and incidence of diabetes are likely higher in developing countries than currently documented due to undiagnosed diabetes. Studies conducted in Africa estimated that 18% of adult diabetes patients in Libya and 75% of adult diabetes patients in Tunisia were undiagnosed.^68^ Generally, the incidence of T2D continues to increase globally but at a steadier rate than in previous decades - this statement could be incorrect due to the uncertain global prevalence and incidence of undiagnosed diabetes.^1^ The rise in the prevalence and incidence of T2D throughout the 20^th^ and 21^st^ centuries globally currently classifies this disease as a global pandemic; the rise in this increase is generally thought to be due to an increase in the percentage of the global population being overweight accompanied by individuals adopting more sedentary lifestyles.^28^

### Aetiology

Many risk factors have been identified for T2D.^3^,^69^ The main risk factor is obesity as being obese can increase the risk of developing T2D by 90-fold, and the majority of patients are overweight or obese.^9^,^15^ T2D risk is positively correlated with increasing BMI, and the risk rises exponentially with increasing BMI above 30.^15^ In Western countries, it has been estimated that ~50% of T2D patients have a BMI of >30 and 30–40% have a BMI of 25–30.^70^ However, in some Asian countries, ~50% of patients are not overweight.^34^,^35^ Surprisingly, underweight T2D patients have even been reported.^71^,^72^ Increased deposition of fat in the ectopic regions of the body (particularly visceral fat) also increases T2D risk by more than double.^3^,^73^ Genetics are known to play an important role in T1D but this is also the case for T2D.^37^,^74^ The concordance rate between monozygotic twins with T2D is higher (around 70%) compared with T1D (between 30 and 50%), and additionally, the lifetime risk of individuals for developing T2D with one affected parent is 40% and almost 70% if both parents are affected - these observations suggest that genetics play an important role in T2D susceptibility.^75^,^76^ Concordance rates between dizygotic twins with T2D (between 20 and 30%) are also higher than T1D (around 10%).^7^,^75^,^76^ However, the highest odds ratio reported for a risk locus for T2D is 1.57 – this implies that more currently untested variants underlie T2D susceptibility.^37^ The increased risk of developing T2D for relatives of type 2 diabetic patients may be due to them sharing similar diets and lifestyles as well as genetics.^7^,^77^ One study demonstrated the importance of genetics in T2D susceptibility independently of the diet as there was a higher prevalence of the disease in the twin population compared to the singleton population^77^– both populations had similar average BMI scores (26.1–26.3) with comparable standard deviation values (3.9–4.7). A cohort study found that alcohol and smoking also increased the risk of T2D, even in individuals who were classified as having low-risk diet and physical activity profiles.^78^

Interestingly, longitudinal studies have demonstrated that “psychological stress-related circumstances” (such as stressful working conditions) or mental health problems (such as depression) increase the risk of T2D.^79^ Globally, more males are diagnosed with T2D than females, and 14 million more men were diagnosed with this disease than the number of women in 2013.^80^ Evidence demonstrates that adult men are at higher risk of T2D than women, which is at least partly thought to be due to differential adiposity storage patterns in men.^81^ Studies have shown that men with T2D are more likely to develop CVD, but women with T2D who do develop CVD are more likely to have a worse prognosis, which is thought to be at least partly due to men being more likely to achieve medical targets in T2D (such as desirable plasma glucose control and blood pressure).^80^,^81^ The seasonality of T2D onset is not well studied, but a study conducted in Hungary found that seasonality followed a sinusoidal pattern; the peak month was March, and the trough month was August.^82^ A recent study also found that Chinese individuals born outside of summer had a 9% increased chance of developing adult T2D than individuals born in summer.^83^ Environmental changes over the last few decades could also play a role in T2D aetiology due to the use of pesticides, drugs and food additives in food processing and packaging.^84^ However, minimal evidence exists linking T2D aetiology to altered food processing/packaging in recent decades.^84^ Some environmental pollutants have been shown to alter β-cell function, and the best example is bisphenol A (used in food container manufacturing), which can cause impaired beta-cell function in animals.^85^ However, it has not yet been determined whether prevailing environmental concentrations of these types of compounds can be a risk factor for diabetes.^84^ Associations have been made between certain pathogens and T2D risk: herpes simplex virus type 1 and hepatitis C virus are risk factors for T2D, although it is not clear how this can be mechanistically explained.^86^,^87^ However, it has been established that hepatitis C promotes insulin resistance in the liver, which is thought to increase T2D risk.^88^,^89^ If this is the case, then this implies that T2D manifestation can be initiated by insulin resistance in the liver alone, which therefore suggests that the liver may play a much more important role in T2D aetiology/pathogenesis than is currently thought.

### Pathogenesis

The main focus here is the current and future therapeutic potential of modulation of GLP-1R activity in T2D, but the altered activity of hormones involved in metabolic homeostasis observed in T2D are also discussed, as well as other factors, such as the nervous system and uncoupling protein 2 (UCP2), to highlight the complexity of its pathogenesis.

Typically, T2D does not manifest acutely in individuals but is preceded by an insidious phase of prediabetes.^90^ Prediabetes is characterised by raised blood glucose levels (fasting plasma glucose levels of 6.1–6.9 mmol/L and two hours post glucose ingestion levels between 7.8–11 mmol/L) due to declining islet beta-cell mass and function but not enough to warrant a diagnosis of T2D.^90^,^91^ Patients with prediabetes are asymptomatic but^90^ ~5–10% progress to T2D each year.^90^,^91^ Studies have demonstrated that weight loss and exercise can usually delay progression to T2D, or even prevent T2D from manifesting; lifestyle interventions reduce the risk of T2D progression in 40–70% of adults with prediabetes.^91^,^92^ It has been estimated that ~70% of individuals with persistent prediabetes will eventually develop T2D, and that >470 million people will have prediabetes by 2030.^91^ Progressing to T2D is characterised by blood glucose levels of >7 mmol/L during fasting and a two hours post glucose ingestion reading of >11 mmol/L.^90^,^93^ The consensus in the literature is that T2D clinical manifestation is provoked by peripheral tissue insulin resistance, which is in turn, usually induced by obesity.^6^,^30^,^94^ Obesity is characterised by elevated levels of cytokines and fatty acids, and it is thought that elevated levels of both provoke insulin resistance.^94^ However, it has still not been determined how this occurs mechanistically.^95^ Following the induction of insulin resistance, islet beta-cells can maintain normoglycaemia and metabolic homeostasis by increasing their secretion of insulin and/or by increasing their number.^49^,^96^,^97^ Obesity has been estimated to induce an ~50% increase in islet beta-cell volume due to increased neogenesis.^49^ However, over time islet beta-cells are seemingly unable to compensate for the insulin resistance and their ability to secrete insulin decreases and many islet beta-cells undergo apoptosis, which is thought to be a result of a variety of stressors, such as increased insulin demand, oxidative, endoplasmic reticulum, dyslipidemic, amyloidal, and inflammatory stress.^98^,^99^ The characteristic consequences of beta-cell pathology during T2D include impaired first-phase insulin secretion, ongoing insufficient insulin secretion to promote normolipidaemia (normal triglyceride levels, normal LDL cholesterol, and normal HDL-cholesterol) and normoglycaemia, dysfunctional glucose-sensing, and an increased proportion of proinsulin secretion.^98^

**Figure 3:**
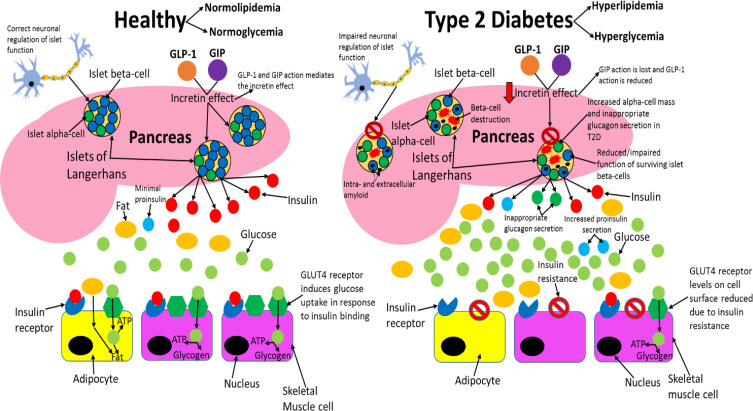
Comparing healthy and type 2 diabetic phenotypes. In healthy individuals, insulin is produced and secreted by beta-cells in the islets of Langerhans (found in the pancreas) when blood glucose levels are above 5mM. Beta-cells are the most abundant cell type in the islets (~70%) and alpha-cells (responsible for glucagon secretion) are the second most abundant (~20%). Insulin then binds to the insulin receptor (IR), which allows uptake of glucose into tissues by inducing translocation of GLUT4 receptors from intracellular vesicles to the plasma membrane. GLUT4 is primarily found in adipose tissue as well as skeletal and cardiac muscle. Glucose is then transported into the cell by GLUT4 from the bloodstream and catabolised in the cell for ATP production, which provides the fuel for intracellular processes, or glucose can be converted to either glycogen or fat for fuel storage after uptake. In individuals with T2D, many islet beta-cells have undergone apoptosis and the function of the surviving cells is impaired, which results in markedly reduced insulin levels in circulation. Additionally, peripheral tissue insulin resistance impairs the action of insulin, resulting in reduced uptake of glucose from circulation, as a result of decreased GLUT4 translocation to the membrane. Reduced insulin levels and action result in hyperglycaemia and hyperlipidaemia, and subsequent T2D associated symptoms manifest in the patient. Inappropriate glucagon secretion, diminished incretin hormone action, increased proinsulin secretion, impaired pancreatic islet neural regulation, and islet amyloid deposition are also characteristic of T2D. This figure and information in its legend are with data adapted from these studies.^105^,^106^,^111^,^326–328^

During prediabetes, pancreatic beta-cell number and function decline slowly, usually over a few years before T2D manifests.^91^ It has been reported that the decline in islet beta-cell function can begin an average of 12 years before T2D diagnosis.^49^ Interestingly, there have been reports of individuals who do not progress to T1D for >10 years despite persistent islet autoimmunity (slow progressors).^100^ This demonstrates that the rate of islet beta-cell death can vary greatly between individuals before T1D or T2D diagnosis, enabling some individuals to remain disease-free for longer, possibly by similar mechanisms. The gradual decline in islet beta-cell number and function results in insulin levels becoming too low to promote metabolic homeostasis and T2D results.^49^,^91^ Hyperproinsulinemia has been reported in both T2D patients and individuals with prediabetes, suggesting that defective islet beta-cell insulin processing is integral to the early stages of disease pathology.^101^,^102^ In healthy subjects, proinsulin accounts for up to 20% of the insulin levels in circulation, but in T2D this value can reach up to 50% and total proinsulin levels have shown to be higher in T2D patients than healthy controls, which suggests that dysfunctional processing and secretion of insulin by the remaining islet beta-cell population further contributes to the decreased insulin levels observed in this disease.^101^,^103^ Symptoms of T2D manifest when insulin levels become too low to prevent hyperglycaemia, which includes dehydration, excessive thirst, increased susceptibility to infection, excessive urination, lethargy and blurred vision.^3^,^47^ Chronic superphysiological glucose concentrations also negatively affect the ability of islet beta-cells to secrete insulin, which further worsens hyperglycaemia and promotes T2D.^104^ Peripheral tissues dependent on insulin to uptake nutrients from circulation can no longer do so to the same extent as before T2D.^47^,^105^ Hence, peripheral tissues adapt to rely on fat and catabolism of intracellular stored macromolecules, such as proteins, to generate the ATP they need.^3^,^32^ This results in weight loss (due to the breaking down of macromolecules), excessive eating and lethargy.^3^,^32^,^105^,^106^ Figure 3 compares healthy and type 2 diabetic phenotypes.

It has been demonstrated that the insulin released during the early and late postprandial period by T2D patients is ~25 and 40%, respectively, of that produced by healthy individuals with a similar BMI.^107^ Fasting insulin levels in T2D patients are ~50% of that observed in healthy subjects with a similar BMI,^107^ demonstrating that T2D has a more significant effect on impairing the insulin response during the postprandial period in the early stages of the disease. Interestingly, insulin levels are higher in diabesity patients than in normal-weight T2D subjects,^21^ implying that the islet beta-cell pathology is more severe in the latter group and that peripheral tissue insulin resistance contributes more to the disease phenotype in overweight T2D patients. Although it is known that the postprandial insulin response is similarly impaired in overweight and normal-weight T2D patients (determined by measuring the increase in insulin levels in comparison to fasting levels), absolute insulin levels are higher in overweight patients compared to disease-free normal-weight subjects, during both fasting and the postprandial period,^21^ which strongly suggests that in overweight T2D subjects peripheral tissue insulin resistance is central to the clinical manifestation of this disease. T1D is more “severe” than T2D, as ~90% of beta-cells are destroyed by autoimmunity, which results in vastly reduced insulin levels.^108^ Insulin treatment is essential for survival in T1D, as the absolute insulin deficiency results in the induction of ketoacidosis in an attempt to provide tissues dependent on insulin with adequate fuel.^106^ Ketoacidosis is necessary during starvation to provide fuel to tissues when there is no adequate nutrient supply.^109^ However, prolonged ketoacidosis is fatal due to the lowering of blood pH, which is why T1D is considered to be a deadlier form of diabetes than type 2, although a subset of T2D patients can develop ketoacidosis.^106^,^110^ Although ketoacidosis is largely considered to be a pathological response, fatality is inevitable under these circumstances, as impaired ATP production is fatal.^32^,^106^,^111^

It has been firmly established that T2D is caused by the dysfunctional exocrine function of the pancreas, and peripheral insulin resistance is a requisite precursor for the development of this disease.^105^,^112^ Skeletal muscle insulin resistance has been associated with intracellular lipid accumulation.^94^ However, endurance-trained insulin-sensitive athletes may have as much, if not more, lipid content in their skeletal muscles as insulin-resistant T2D patients.^113^,^114^ Thus, it seems that a high rate of ATP turnover in athletes prevents skeletal muscle insulin resistance, but a decreased demand for ATP in typically sedentary obese individuals with and without T2D induces insulin resistance.^94^ It is reasonable to assume that insulin resistance in obese individuals without T2D may be desirable in skeletal muscle that has an abundance of stored nutrients and a low rate of ATP turnover, as the tissue’s ATP demands are met and hypertrophy would result from any further nutrient uptake. However, it is correct to state that peripheral tissue insulin resistance is pathological in T2D as it contributes to the disease phenotype.^30^,^47^ The insulin resistance in peripheral and hepatic tissues is present in obese and non-obese T2D patients, but not to the same extent.^115^ Studies have shown that BMI is correlated to the degree of insulin resistance in Korean patients, even within the non-obese range.^116^ Weight loss has been shown to decrease insulin resistance in both obese T2D patients and obese T2D-free individuals, suggesting that insulin resistance is related to increased nutrient storage.^117^ Interestingly, insulin resistance is also observed in a proportion of T2D-free individuals within the normal weight range (BMI>18.5–25) and is reasonable to assume that this is pathological as, in theory, peripheral tissues are not at risk of hypertrophy due to insulin action and require continual nutrient influx to maintain ATP supply to meet their metabolic demands, as these individuals are not overweight and do not have an abundance of stored nutrients within their tissues.^23^,^118^ One study demonstrated that normalisation of both beta-cell function and hepatic insulin sensitivity in T2D patients was achieved by 8 weeks of dietary energy restriction alone (600 kcal a day), accompanied by decreased pancreatic and liver triacylglycerol stores.^119^ After cessation of the low-calorie diet and returning to a normal diet for 12 weeks, 7 out of 10 participants remained in remission. Given that lipid accumulation both in and around the pancreas is associated with beta-cell pathology and confers T2D risk,^120^ the observation that T2D remission was accompanied by a decrease in pancreatic fat content suggests that increased pancreatic fat storage is an important component of T2D aetiology/pathogenesis. In healthy individuals, insulin sensitivity varies naturally, which is thought to be beneficial for promoting metabolic homeostasis.^121–123^ The general consensus in the literature is that chronic insulin resistance, induced by obesity, is thought to be pathological overall in the metabolic syndrome, which is a syndrome characterised by a range of disorders that collectively predispose risk to T2D and cardiovascular pathology.^94^

A study examined pancreatic tissue from 124 autopsies: 91 obese cases (BMI >27kg/m^2^; 41 with T2D, 15 with impaired fasting glucose [IFG], and 35 non-diabetic subjects) and 33 lean cases (BMI <25kg/m^2^; 16 types 2 diabetic and 17 non-diabetic subjects.^124^ Obese humans with IFG and T2D had a 43 and 60%, respectively, decrease in islet beta-cell volume compared to obese nondiabetic individuals. This decrease in islet beta-cell volume in obese T2D patients was found to be due to a decreased number of islet beta-cells, and interestingly the volumes of these cells were found to be similar between non-diabetic obese and diabetic obese individuals. The frequency of islet beta-cell replication was found to be similar between obese nondiabetic and T2D patients. However, it was estimated that there was an ~3-fold increase in apoptosis in obese T2D individuals. The majority of diabesity cases were found to have islet amyloid in their islets (~80%), whereas only ~10% of obese individuals without T2D had amyloid deposits. Amyloid deposits had a similar prevalence in IFG obese individuals to that observed in T2D-free obese individuals. Islet beta-cell dysfunction in diabesity patients is therefore attributed to increased rates of apoptosis and amyloid deposition, and not to rates of replication and new islet formation as this is similar to non-diabetic controls.

Currently, why individuals of normal weight develop T2D is largely elusive, but pathogenesis appears to be similar to diabesity patients, although there are some striking differences according to the study conducted in 2003.^124^ Islet beta-cell mass was shown to be decreased in lean T2D patients by ~41% in comparison to lean T2D-free controls, due to a decreased number of these cells. Islet amyloid was present in 88% of lean T2D patients but only 13% of lean healthy controls. New islet formation and beta-cell replication were similar between lean T2D patients and lean T2D-free individuals, but the frequency of beta-cell apoptosis was increased by 10-fold in lean T2D patients. Hence, lean T2D is also characterised by an increased rate of beta-cell apoptosis with a normal, but too slow a replication rate of these cells to restore their numbers to normal levels. It is not known if islet amyloid deposits are produced due to the chronic hyperglycaemia associated with this disease. It is also not clear if these deposits affect the prognosis of T2D.^124^,^125^

What has become clear is that islet amyloid deposits are very likely to be directly involved in the pathogenesis of T2D, although it has not been well established how mechanistically.^124^,^126^,^127^ Islet amyloid polypeptide (IAPP) (more commonly known as amylin) is co-secreted with insulin from islet beta-cells in healthy individuals during the fed state and acts as an anti-hyperglycaemic agent by decreasing gastric motility, promoting satiety and inhibiting glucagon secretion.^128^ The amylin analogue pramlintide is used for T1D and T2D treatment adjunct with insulin therapy in the USA if patients are not achieving adequate glucose control despite optimal insulin therapy.^129^ Virtually all T2D patients have IAPP-containing plaques in their islets.^128^ IAPP appearance was first noticed in the 1980s to correlate with hyperglycaemia in primates.^130^ Several lines of transgenic mice have been generated which produce human IAPP, and they were shown to have islet amyloid accumulation, increased beta-cell apoptosis, decreased beta-cell mass, impaired glucose tolerance, and some developed diabetes.^128^ The current consensus in the literature is that extracellular plaques of IAPP are not causative of beta-cell pathology, but rather intracellular oligomers of this protein observed in T2D patients (but not in T2D-free individuals) produce the cytotoxic effects.^128^,^131^ It has been elucidated that beta-cells are usually protected from IAPP oligomers by autophagy processes, so it seems that these processes are defective in prediabetes and T2D, which is likely pivotal for the pathogenesis of T2D.^128^,^132^ Both T1D and T2D manifest due to beta-cell apoptosis and the activated apoptotic pathways are thought to share similarities, but the aetiology of apoptosis is different between the two diseases.^133^

Although obesity is a major risk factor for T2D, paradoxically overweight and obese patients have been reported to have had a lower mortality rate than normal-weight patients due to cardiovascular pathology associated with T2D and this phenomenon was termed “the obesity paradox”.^134^,^135^ Furthermore, weight loss has been reported to increase mortality and morbidity in T2D and cardiovascular co-morbidity patients.^136^ Additionally, a study found that the risk of amputation in non-elderly diabetic men decreased with increasing body weight.^137^ Peripheral and hepatic insulin resistance is present in obese and non-obese T2D patients, but not to the same extent in non-obese subjects.^115^ Studies have shown that BMI is correlated to the degree of insulin resistance in Korean patients, even within the non-obese range.^116^ Interestingly, a study from India found that fasting and postprandial plasma glucose levels were higher in T2D patients with a BMI <18.5.^71^ The aetiology of T2D patients with a BMI of under 25 is thought to be largely attributed to beta-cell dysfunction, which is thought to be greater than that observed in obese patients.^71^ A study conducted in Chicago found that glycaemic control was worse in T2D patients with a BMI of 18–25 than in individuals with a BMI of over 25, but these patients had lower insulin resistance.^138^ The study found that smoking and alcoholism confer an increased risk for non-obese T2D than obese T2D, chronic alcoholism induces islet beta-cell dysfunction and apoptosis.^139^ Strikingly, T2D has been shown to manifest in non-overweight individuals in the absence of insulin resistance, although this is thought to be rare.^140^ This implies that pancreatic exocrine dysfunction can induce T2D independent of both insulin resistance and a BMI>25. Whether or not weight loss in T2D patients with a BMI of under 25 improves prognosis has not been determined.^71^ It has been well established that Asians are at increased risk of non-overweight T2D. In Japan, more than 50% of patients have a BMI of under 25, but they have more abdominal fat at the equivalent BMI to Caucasians.^35^,^71^

T2D is not only attributed to beta-cell dysfunction but also alpha-cell dysfunction.^141^,^142^ Pathologically high plasma concentrations of glucagon are observed in T1D, advanced T2D, and diabetic ketoacidosis.^142^ It is well established that islet alpha-cell populations increase in T1D, whereas in T2D, patients can have alpha-cell numbers increased or unaltered.^141^ Paradoxically, T2D patients have increased levels of glucagon during the postprandial period, leading to augmentation of postprandial hyperglycaemia associated with impaired insulin secretion.^142^,^143^ Hence, it is correct to state that the behaviour of diabetic islet alpha-cells is different for healthy individuals, as an abundance of nutrients during the postprandial period prevents glucagon secretion in healthy individuals by raising ATP levels (as discussed earlier in this review).^144^ Therefore, in T2D, the islet alpha-cells must have altered biochemical pathways inducing altered mechanisms of glucagon secretion. Studies have estimated that postprandial hyperglucagonemia may contribute to ~50% of the pathological increase in plasma glucose levels after glucose ingestion.^145–147^ Interestingly, fasting hyperglucagonemia has been reported in some T2D patients with moderate glycaemic control, and glucagon levels are ~50% higher in diabetic subjects.^146^,^148^ The dysfunctional glucagon secretion in T2D could be due to the decreased insulin levels, as insulin suppresses alpha-cell function.^105^,^149^ However, islet alpha-cells should have their exocytotic function suppressed during the fed state, as intracellular ATP levels should be high as a result.^144^ However, studies have demonstrated that blocking insulin signalling in islet alpha-cells in mice results in defective suppression of the fed state on glucagon secretion, highlighting the pivotal role of insulin in the regulation of the alpha-cell.^105^,^149^

**Figure 4:**
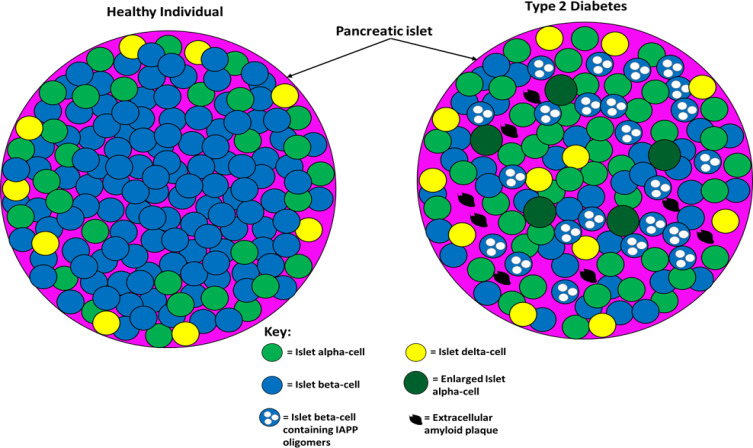
The difference between pancreatic islet architecture in healthy individuals and individuals with T2D. In healthy individuals, beta-cells are situated centrally and peripherally and are the most abundant cell type (~70%). Non-beta cells are found in the periphery of the islets and constitute ~30% of the cell population (20% alpha-cell and 10% other cell types). Islet architecture is altered in T2D with a greatly diminished population of beta-cells, more alpha-cells, more delta-cells, migration of alpha- and delta-cells into the centre, extracellular amyloid plaque deposits, intracellular IAPP oligomers in beta-cells, and enlarged alpha-cells. The altered architecture in T2D produces different intra-islet paracrine signalling which impairs metabolic homeostasis. This figure and information in its legend are with data adapted from Brereton et al.^141^

Interestingly though, dysregulated glucagon secretion is not significant in adolescents with T2D.^150^ There is some evidence that the liver is more sensitive to glucagon in T2D patients, further exacerbating the hyperglycaemia, although there have been conflicting findings with this observation.^142^ Both T1D and T2D induce major changes in the structure of animal and human pancreatic islets.^141^ The changes in the islets of T2D patients have been postulated to induce altered signalling within the islets, which contributes to the phenotype of this disease.^141^ The increase in alpha-cell mass likely results in increased alpha–alpha cell contacts at the expense of beta–beta cell contacts, which may alter intra-islet signalling.^151^ The amyloid deposits in the islets have also been postulated to alter intra-islet signalling.^124^ Islet alpha-cells are usually found in the periphery of the islets, but in diabetes, many of the alpha-cells are found in the centre as well; in rodent models, this can be reversed by restoring normoglycaemia.^141^,^152^ The proportion of delta–delta and delta–alpha cell contacts also increases, likely as a result of the beta-cell destruction in T2D.^151^ In T2D rodent models, delta-cells were reported to migrate from the periphery of the islets to the centre.^152^ How the morphology, migration and number of islet gamma- and epsilon-cells are altered in T2D is not well studied.^141^ Although there have been reports suggesting that the delta-cell number is increased in diabetes, other studies suggested that the number of these cells was unaltered in patients’ islets.^153^,^154^ Interestingly, a recent study found that the delta-cell number and volume were decreased in baboons with impaired fasting glucose by ~41%, and this was due to apoptosis.^155^ Evidence is emerging that a subset of islet beta-cells may undergo conversion into other endocrine islet cell types in T2D, which may account for the increased alpha-cell population observed in some T2D patients and their distorted insulin-to-glucagon ratios.^156^ Figure 4 compares pancreatic islet architecture between healthy and type 2 diabetic individuals.

The incretin effect is greatly diminished in T2D where GLP-1 and GIP account for <20% of the insulin release after glucose ingestion in patients.^157^ The current consensus in the literature is that GLP-1 and GIP circulation levels are comparable between T2D patients and nondiabetic controls in response to nutrient challenges.^158^ Hence, the diminished incretin effect is due to dysfunctional beta-cells.^159^ GLP-1 infusions can normalise blood glucose levels, and GLP-1 analogues are used to treat T2D.^3^ However, GIP action is lost in T2D and even infusions of GIP that result in supraphysiological concentrations fail to elicit a significant insulin secretory response in T2D patients.^160^ Studies have demonstrated that intensive insulin administration treatment results in improved insulin secretion from islet beta-cells after GIP infusion.^161^,^162^ It has been established that hyperglycaemia induces down-regulation of GIPR expression in beta-cells, so it is plausible that in this study GIPR expression may have been upregulated in these cells due to reducing plasma glucose levels.^159^ Therefore, although it is clear that GIP action is lost in T2D it could still have future therapeutic potential for treating T2D. GIP action in prediabetes has not been established to date, so it is currently not clear if impaired GIP action is involved in T2D pathogenesis or manifests post-diagnosis.

**Figure 5:**
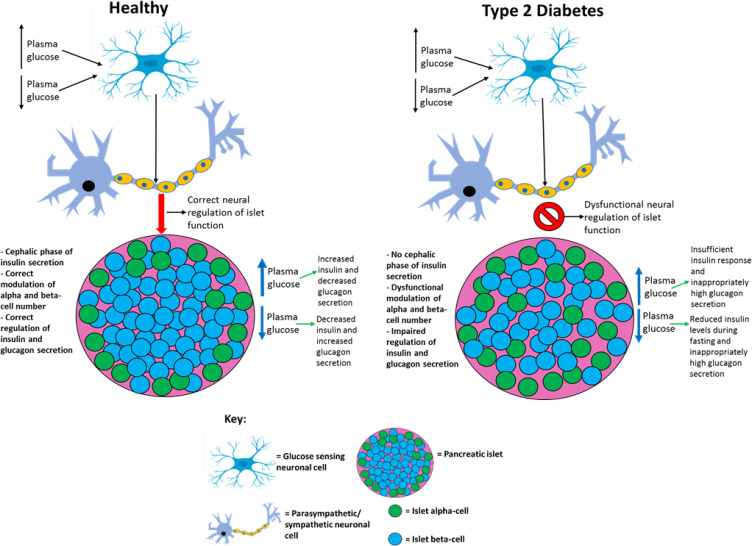
Comparing the differential neural regulation of pancreatic islets in healthy and type 2 diabetic individuals. T2D patients do not exhibit the cephalic phase of insulin secretion and based on the findings from studies it is possible that dysfunctional neuronal regulation of pancreatic islets contributes to the impaired GSIS, hyperglucagonemia, and decreased beta-cell proliferation and mass observed in this disease. This figure and information in its legend are with data adapted from these studies.^163^,^166^

Additionally, pancreatic neuronal activity is also impaired in T2D.^163^ The pancreas is richly innervated by the sympathetic and parasympathetic nervous system.^164^ The cephalic phase of insulin secretion is lost in T2D and glucagon secretion is not suppressed during the fed state.^163^ This implies that if the nervous system does suppress glucagon secretion in an insulin-independent manner during the fed state in healthy individuals then this is absent in T2D. The parasympathetic activity also plays a role in stimulating beta-cell proliferation in adult animals, which is at least partly regulated by acetylcholine binding to muscarinic receptors on beta-cells.^164^,^165^ The neuronal activity has also been postulated to modulate the alpha-cell number.^163^ This demonstrates that the nervous system is needed for both islet function and islet-cell proliferation when appropriate. Parasympathetic and sympathetic nervous systems are known to influence endocrine pancreas postnatal development and plasticity in adult animals.^163^ Dysfunctional regulation of the pancreatic islet by the nervous system contributes to the clinical phenotype of T2D, but whether or not this occurs before or after T2D diagnosis remains elusive, although it is reasonable to assume that it does, influencing the altered islet architecture observed in T2D.^141^,^163^ It has been confirmed that glucose-sensing cells in the central nervous system (CNS) are excited by either a rise (glucose excited neurons) or decrease (glucose inhibited neurons) in plasma glucose levels, and it has been postulated that these neurons regulate the sympathetic and parasympathetic branches of the autonomic nervous system, which are known to influence insulin and glucagon secretion.^163^ It is likely that the pathology of these central neurons contributes to the T2D phenotype and may partly, or even exclusively, induce T2D pathogenesis by currently unknown mechanisms. One study clearly demonstrated the importance of the nervous system’s ability to regulate islet beta-cell number and function: GLUT2 neuronal knockout mice exhibited impaired glucose-stimulated insulin secretion (GSIS), hyperglucagonemia, and decreased beta-cell proliferation and mass in comparison to controls.^166^ Importantly, treatment of the control mice with the ganglionic blocker chlorisondamine reduced the proliferation rate of beta-cells by 50%, but in GLUT2 neuronal knockout mice, chlorisondamine did not further reduce the proliferation rate. This study clearly shows that the activity of the neuronal glucose-sensing cells and islet alpha- and beta-cells function synergistically to promote appropriate plasma insulin and glucagon levels – this synergism is known to be dysfunctional in T2D.^163^ Figure 5 summarises the differential neural regulation of the pancreas between healthy and type 2 diabetic individuals.

The notion that glucolipotoxicity conditions in circulation (associated with obesity) induces islet beta-cell dysfunction has been an attractive area of research.^167^ A number of in vitro studies, using insulin-secreting cells and isolated islets, have found that exposure of islet beta-cells to glucolipotoxicity conditions results in impaired GSIS, impaired insulin expression, and cell death by apoptosis.^167^,^168^ Importantly, several of these studies have demonstrated that fatty acids do not induce beta-cell dysfunction in the absence of elevated glucose, highlighting that fatty acids and glucose are not cytotoxic independently.^168^,^169^ The application of these experiments to in vivo situations is questionable as it has been demonstrated that non-diabetic obese subjects exhibit increased insulin secretion and an increase in beta-cell number and mass, and islet amyloid deposits are rare in these individuals as aforementioned in this review. Thus, it is reasonable to assume that glucolipotoxicity diets do not induce beta-cell pathology before the prediabetes phase and pre-T2D diagnosis. However, in vivo studies on healthy rats have demonstrated similar findings to that of in vitro when studying the effects of glucolipotoxicity on insulin secretion.^170^ Given that weight loss and a controlled diet improve glycaemic control in overweight T2D patients, it seems likely that glucolipotoxicity diets worsen the disease phenotype post-diagnosis.^12^ However, attributing T2D pathogenesis to glucolipotoxicity diets does not explain how individuals with a BMI of under 25 develop this disease. To further complicate the issue, data obtained from humans in lipid infusion studies have produced conflicting results as to whether or not glucolipotoxicity conditions impair or boost insulin secretion.^171^

Studies conducted by Affourtit and colleagues attempted to explain mechanistically how glucolipotoxicity conditions might impair GSIS.^172^,^173^ Interestingly, they have found that the impaired GSIS observed during in vitro studies involving exposing pancreatic beta-cell line INS-1E to glucolipotoxicity conditions (palmitate + high glucose) was found not to be dependent on mitochondrial dysfunction.^173^ From the observations of this study, it seems that whatever role glucolipotoxicity may have in impairing GSIS in T2D is disconnected from mitochondrial dysfunction, which is surprising given the role of the mitochondria in ATP and reactive oxygen species (ROS) generation needed for both phases of insulin secretion.^174^,^175^ The concept of “beta-cell rest” to treat T2D has emerged given the observations from both in vitro and in vivo studies.^98^ Isolating islets from diabetic rodents and exposing them to euglycemic conditions for 8–12 h resulted in the return of both normal insulin secretory capacity and beta-cell function.^89^ Furthermore, bariatric surgery (which results in reduced food intake post-surgery) and low-calorie diets can induce T2D remission, which can be viewed as in vivo evidence that reducing demand on the beta-cell (ie, “beta-cell rest”) improves endogenous insulin secretory capacity.^98^ Administering diazoxide (which inhibits endogenous insulin secretion and therefore induces a degree of transient “beta-cell rest”) to T2D patients resulted in a 280 and 500% increase in insulin levels over baseline after stimulation tests in T2D low and high beta-cell reserve groups, respectively.^176^ This study provides in vivo evidence that “beta-cell rest” can at least temporarily allow insulin secretory capacity recovery, and in vitro studies examining the effect of diazoxide on diabetic human islets produced similar findings.^177^ Another in vivo study found comparable effects on improved beta-cell function following short-term continuous subcutaneous insulin infusion in T2D patients that were uncontrolled by conventional oral therapies.^88^ This resulted in near euglycemia being achieved in the participants and glycaemic control was improved after cessation of insulin infusion. Islet beta-cell function tests conducted 2 days after cessation of insulin infusion revealed that the peak insulin response was notably improved. Reducing demand on the islet beta-cells by improving insulin sensitivity through weight loss and/or pharmacological means has been reported to improve T2D prognosis and delay/prevent pre-diabetic individuals progressing to T2D, suggesting that inducing “beta-cell rest” to even a degree alleviates the pathology that is associated with this disease.^92^,^98^ Despite the evidence generated by both in vitro and in vivo studies supporting that glucolipotoxicity impairs beta-cell function, these findings likely do not apply to non-obese/overweight diabetic individuals.

UCP2 is a protein present in the beta-cell mitochondria inner membrane, which was postulated to dampen GSIS by dissipating mitochondrial proton motive force as heat (due to its analogy with UCP1), thereby reducing the efficiency of ATP production. Approximately 20% of the proton leak in beta-cells is thought to occur due to the presence of UCP2 .^178^,^179^ The detection of this protein in islet beta-cells is puzzling as sufficient ATP levels are vital for the correct function of these cells, and studies have shown that overexpression of UCP2 in isolated beta-cells impairs GSIS.^180–182^ It has been shown that a high proton motive force (PMF) results in the production of ROS, and as UCP2 activity can lower the PMF by increasing proton leak, it can then lower intracellular ROS, as well as increased production of ROS, is thought to be at least partly dependent on an increased mitochondrial PMF in islet beta-cells.^180^,^183–185^ As ROS are largely thought to be cytotoxic molecules, it was assumed that the fact UCP2 attenuates ROS production could mean that this protein has a protective role in the cell.^32^,^185^,^186^ This notion had led to a debate as to whether or not the presence of UCP2 in these cells is pathological given its ability to decrease ATP production or beneficial given its ability to regulate levels of ROS.^179^ However, it has been demonstrated that UCP2 does not appear to regulate GSIS, as palmitate + high glucose impaired GSIS to the same extent in INS-1E cells with and without UCP2 (UCP2 was knocked down via RNAi).^187^ UCP2 knockdown was shown to only minimally and not significantly increase the coupling efficiency (<10%) when INS-1E cells were exposed to 28mM glucose and palmitate. Oddly, it was found that palmitate with 28 mM glucose dampened the coupling efficiency in UCP2 depleted cells more than in UCP2 positive cells. Based on these findings, it appears that UCP2 is not involved in either GSIS or regulation of the efficiency of mitochondrial energy transduction in INS-1E cells in vitro.

**Figure 6:**
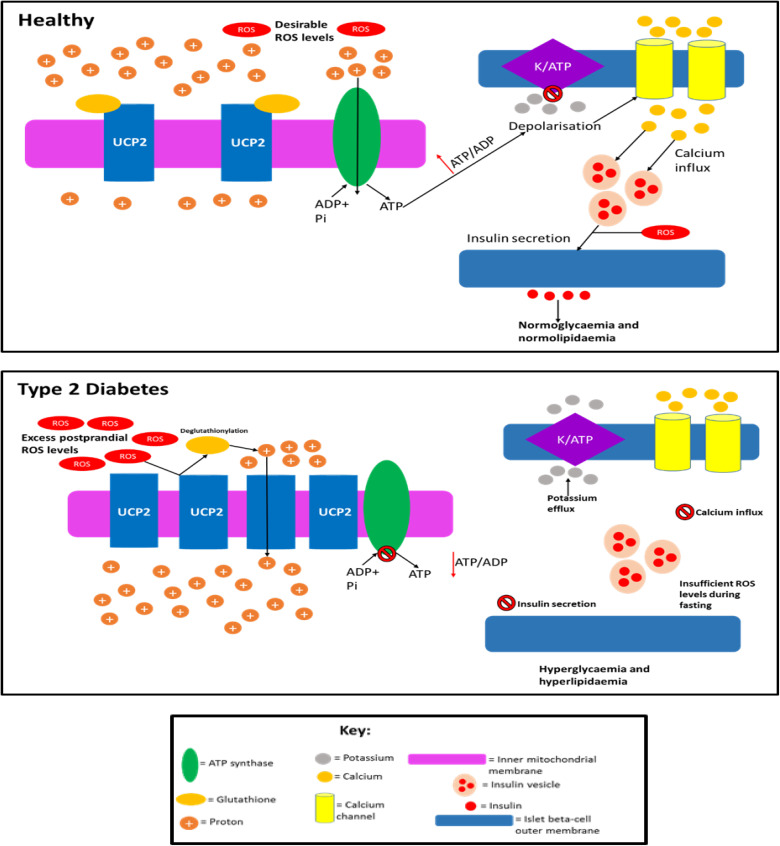
Speculation of how UCP2 activity and quantity is altered in T2D. In healthy individuals: UCP2 levels are basal, UCP2 is glutathionylated, ROS levels are low enough so that they do not cause cell damage but the levels are sufficient so that these molecules can amplify insulin secretion, and proton leak exists (~55% of islet beta cells’ metabolism is wasted due to proton leak which is not caused by UCP2) but is minimal, so enough of the protons can move down the gradient through ATP synthase to generate sufficient ATP levels. In T2D, UCP2 is deglutathionylated (due to increased ROS levels) and upregulated allowing for much greater proton leak. Consequently, the majority of the protons prematurely leak through the inner mitochondrial membrane and do not pass through ATP synthase, although there is likely a period before the UCP2 deglutathionylation where ATP production occurs, it is unlikely that enough ATP is generated during this time to promote adequate insulin secretion. Hence, overall ATP production is impaired in the cell resulting in the inadequate insulin secretion observed in T2D during both the postprandial period and fasting. ROS levels are likely too high (leading to cytotoxic damage) during the postprandial period as a result of severe nutrient oversupply to islet beta-cells, due to the enhanced hyperglycaemia and hyperlipidaemia in T2D during this period. The excess ROS levels result in UCP2 deglutathionylation and a subsequent dissipated PMF, meaning that ROS formation during fasting is impaired and insulin secretion is not amplified by ROS to alleviate the chronic hyperglycaemia and hyperlipidaemia in fasting T2D subjects. Although UCP2 may become glutathionylated upon ROS levels decreasing during fasting, this likely reverses during subsequent feeding as ROS levels once again become undesirably high, restarting this cycle. This figure and information in its legend are with data adapted from these studies.^32^,^175^,^178^,^179^,^189^,^190^,^329^,^330^

It has been postulated that UCP2 may not be an uncoupling protein despite its analogy with UCP1, but it likely functions as a carbon skeleton exporter from the mitochondrial matrix, and there is experimental evidence for this.^188^ To further complicate the role of UCP2 in GSIS, in vivo studies have found that global UCP2 knockout mice exhibit either improved glucose tolerance and GSIS, or unaltered glucose tolerance and impaired GSIS depending on their genetic background.^179^,^187^ Beta-cell specific ablation of UCP2 produces glucose-intolerant mice that have increased proton gradients across their inner mitochondrial membranes (suggesting that this protein does mediate premature proton leak), but oddly, ATP levels and respiration rates are unaltered.^184^ The enhancement of GSIS in the beta-cells of these mice was suggested to be due to the increased ROS levels associated with UCP2 knockout. The glucose intolerance observed in these mice was thought to be due to a greater alpha-cell area and higher glucagon content in the islets, and glucagon was secreted by alpha-cells even during high plasma glucose, suggesting that UCP2 presence in beta-cells is necessary to maintain appropriate alpha-cell behaviour during the fed state. Affourtit and colleagues have demonstrated that mitochondrial function plays an important role in cell viability, as palmitate-induced mitochondrial superoxide formation results in reduced viability; this can be prevented by co-exposure to palmitoleate (an unsaturated fatty acid).^172^ Hence, mitochondrial activity during glucolipotoxicity may play an important role in beta-cell survival in vitro and in vivo. A critical gap in UCP2 research is that studies have not taken into account the glutathionylation state of this protein.^189^ Glutathionylation and deglutathionylation are thought to deactivate and activate proton leak, respectively.^189^,^190^ There is evidence that this post-translational modification is regulated by intracellular ROS levels (increased ROS causes deglutathionylation and decreased ROS does the opposite); hence, it is now relatively well established mechanistically how ROS levels can regulate themselves in these cells.^189^,^190^ It is important that future studies examining UCP2 activity and levels also monitor glutathionylation status as well, which could explain any unexpected phenotypes. Currently, the role of UCP2 in healthy individuals and T2D patients is unclear, but if this protein behaves pathologically in T2D by inducing decreased ATP production, then this could explain the dysfunctional insulin secretion observed. Figure 6 proposes how UCP2 activity and quantity alter in T2D.

Glucolipotoxicity conditions induce beta-cell dysfunction in vitro but it is not clear if this is the case in vivo due to the reasons aforementioned. Additionally, GSIS defects in vitro do not appear to be attributable to the mitochondria, but this has not been confirmed in vivo, and the role of UCP2 and its glutathionylation state in GSIS in vivo is currently a source of confusion.

## Extrapancreatic Tissues Involvement in T2D: Aetiology/Pathogenesis and Post-Diagnosis Complications

Whilst it is firmly established that pathological pancreatic function and peripheral tissue insulin resistance are central to T2D aetiology/pathogenesis, there is increasing evidence that other extrapancreatic pathologies may contribute.^30^,^191^,^192^ The diabetic phenotype includes systemic pathology, such as in the CVS, skeletal muscle, nervous system (central and peripheral), skin, kidneys and gastrointestinal tract (GIT), which further exacerbates the disease phenotype and/or induces post-diagnosis complications.^30^,^193–195^ However, research evidence over the last few decades suggests that some of these pathologies manifest during the prediabetic phase and is possibly therefore involved in both the disease pathogenesis and aetiology.^191^,^192^ For example, skin and liver pathology can manifest during the prediabetic stage, and even in individuals with metabolic syndrome before islet beta-cell dysfunction occurs.^191^,^196^

It is firmly established that chronic insulin resistance in skeletal muscle of people with T2D contributes to the diabetic phenotype and results in poor exercise tolerance.^3^,^197^ However, the altered metabolism of skeletal muscle in patients results in a reduced capacity to oxidise fat and promote fat storage, which further exacerbates the disease phenotype.^192^ This behaviour of skeletal muscle is more pronounced in T2D as diabesity patients have a reduced ability to oxidise fat than obese-matched controls during exercise.^192^,^198^ Impaired energy utilisation results in decreased exercise tolerance and increased fat storage, which further promotes insulin resistance.^192^ Interestingly, this pathological behaviour of skeletal muscle has also been detected in individuals with prediabetes, suggesting that these alterations may play a role in the development of both prediabetes and T2D.^192^,^199^ However, contradictory to this, some studies have shown that fat oxidation is increased in the muscle of T2D patients.^200^ Additionally, metabolic inflexibility is also characteristic of T2D patients, whereby patients’ ability to switch from lipid to carbohydrate oxidation during insulin stimulation is impaired, which also further contributes to impaired exercise tolerance.^192^ Obese normoglycemic individuals were shown to have better metabolic flexibility than weight-matched T2D patients.^201^ However, studies have found that metabolic inflexibility of glucose is largely due to defective glucose transport,^201^,^202^ which suggests that skeletal muscle is unable to promote sufficient insulin secretion from type 2 diabetic islet-beta cells via any signalling mechanisms, in which it likely attempts to achieve given its need for increased glucose uptake.^3^,^197^ Additionally, given that metabolic inflexibility can be improved by weight loss and exercise, this further supports the notion that impaired glucose uptake largely accounts for this phenomenon, as these activities result in improved insulin sensitivity.^203^

The phenomenon of metabolic inflexibility has also been observed in individuals with prediabetes, implying that this may play a role in the aetiology/pathogenesis of both prediabetes and T2D.^204^ Mild muscle atrophy is common in middle-aged T2D patients and becomes more significant during older age and diabetic neuropathy.^205^,^206^ Although inflammation and chronic insulin resistance are thought to promote muscle atrophy in T2D patients,^206^ it is reasonable to assume that the impaired energy utilisation promotes the degradation of intracellular proteins to yield sufficient ATP levels, which would contribute to the observed muscle atrophy. Evidence is also emerging that type 2 diabetic muscle may have detrimental effects on beta-cell function via secretion of myokines into circulation: myokine profiles differ between T2D patients and controls.^207^ TNFα secretion from T2D subjects was higher than healthy controls,^207^,^208^ and one in vitro study has shown that this myokine impairs beta-cell function.^208^ Decreased numbers of mitochondria have been observed in the muscle cells of T2D patients, which has created the notion that a decreased population of mitochondria induces insulin resistance.^200^ However, rodent studies have found that an increase in mitochondrial biogenesis occurs concomitantly with the development of muscle insulin resistance, implying that this notion is incorrect.^209^,^210^ This increase in mitochondria is postulated to be an early, transient event that is lost as the insulin resistance progresses.^211^ Furthermore, decreasing the mitochondrial population or disrupting mitochondrial function increases basal and insulin-stimulated glucose uptake into rodent skeletal muscle.^200^

Decreased insulin secretion is known to result in increased hepatic glucose production, which contributes to hyperglycemia, but type 2 diabetic livers also exhibit hepatic insulin resistance, which further increases glucose production during the fasting and postprandial period, giving the liver a direct role in contributing to the disease phenotype.^192^,^212^ Interestingly, insulin-induced suppression of gluconeogenesis and glycogenolysis has also been shown to be impaired in individuals with prediabetes, suggesting that altered liver function is an important component during the initial stages of T2D pathology.^191^ The diabetic phenotype promotes fat storage in the liver due to elevated fatty acid levels in circulation and ~70% of T2D patients develop non-alcoholic fatty liver disease (NAFLD).^213^,^214^ Elevated plasma lipid levels in T2D result in increased delivery of fatty acids to the liver, which is largely caused by reduced suppression of lipolysis in adipose tissue due to decreased insulin action/secretion in diabetes.^213^ NAFLD is an established risk factor for CVD and so increases the risk of cardiovascular complications in T2D patients.^214^,^215^ T2D also increases the risk of NAFLD progressing to non-alcoholic steatohepatitis and subsequent liver cirrhosis.^214^ The increased fat storage in the liver of T2D patients further promotes hepatic insulin resistance and induces impaired hepatic fatty acid oxidation, which in turn, further increases hepatic fat storage and exacerbation of insulin resistance, which worsens hyperglycemia as a result.^213^,^216^

Proinflammatory cytokines, produced by adipose tissue as a consequence of obesity, are known to be cytotoxic to beta-cells, and they likely contribute to T2D pathogenesis and islet dysfunction post-diagnosis.^99^,^217^ There is also evidence that these cytokines play a role in the induction of insulin resistance.^218^ Thus, the adipose tissue also likely contributes to T2D aetiology/pathogenesis, but to what extent remains currently unknown.^94^ The reduced insulin levels also result in increased lipolysis and decreased fat storage in adipocytes, resulting in hyperlipidaemia and increased deposition of fat into other tissues, such as skeletal muscle and liver.^219^,^220^ There is also evidence that altered gut microbiota profiles observed in T2D patients and overweight subjects also promote systemic inflammation and proinflammatory cytokine production, which is thought to encourage the disease pathogenesis and contribute to the disease phenotype post-diagnosis.^221^ T2D patients exhibit alterations in bone behaviour, which is believed to increase the risk of fracture and promote bone fragility: older adults with T2D have up to an 80% increase in the risk of extremity fracture.^222^ Decreased levels of the parathyroid hormone have been seen in T2D, indicating decreased bone turnover, and increased levels of sclerostin in patients also indicate inhibited bone formation.^222–224^ Decreased insulin-like growth factor (IGF)-1 levels in T2D also likely results in decreased bone formation in T2D.^222–224^

Evidence is now emerging that T2D induces premature ageing of the CNS, as some (but not all) studies have found that patients exhibit impaired cognitive performance and electrophysiological defects in the hippocampus, as well as pathological brain morphological abnormalities – all of which are reminiscent of the changes observed during normal ageing.^225^ Interestingly, GLP-1R is downregulated in the hypothalamus of T2D patients in comparison to healthy controls, suggesting that the reduced ability of GLP-1 to induce satiety may contribute to the dysfunctional feeding behaviours and metabolic homeostasis observed in these patients.^226^ Whether or not this downregulation occurs before or after T2D diagnosis is unknown.

Peripheral neuropathy is a common complication of T2D, and an estimated 50% of patients develop diabetic neuropathy after 25 years of being diagnosed.^227^,^228^ Neuropathy may result from excessive levels of nutrients in circulation (resulting in complications such as oxidative damage) and impaired cardiovascular function (resulting in complications such as hypoxia), discussed in detail elsewhere.^228^ Diabetic neuropathy can have a range of consequences depending on the neurons affected: sensory neuropathy results in numbness or pain, motor neuron neuropathy results in impaired muscle movement and autonomic neuropathy results in dysfunctional regulation of involuntary activities, such as internal organ function.^229^,^230^

A recent study found that activation of preproglucagon (PPG) neurons in the brainstem of rodents reduced basal hepatic glucose production, enhanced intraperitoneal glucose tolerance, and augmented hepatic insulin sensitivity. This suggests that PPG neuron-mediated circuitry has an important physiological role in promoting glycaemic control and insulin sensitivity and that neuronal activity can promote metabolic homeostasis via extrapancreatic methods.^104^ Another recent study found that the activation of cholinergic preganglionic neurons in rodents via the melanocortin-4 receptor agonist lorcaserin reduced hepatic glucose production, increased glucose disposal and improved insulin sensitivity.^129^ Lorcaserin has also been reported to improve glycaemic control and induce weight loss in obese T2D subjects, and this drug was additionally shown to both reduce the risk of prediabetic individuals progressing to T2D and increase the likelihood of these subjects being able to revert to euglycemia.^129^,^231^ Given the likely role of these neurons in promoting metabolic homeostasis in humans, it is likely that PPG and cholinergic preganglionic neuronal activity is impaired or absent in T2D, possibly as a result of the disease phenotype, or dysfunction of these neurons could occur at an early stage and play a role in the disease’s aetiology/pathogenesis.

The diabetic phenotype greatly increases the risk of atherosclerotic plaque formation due to dyslipidaemia in patients, which results in increased plasma levels of the small dense atherogenic form of LDL cholesterol, and these molecules can easily penetrate the arterial wall and promote atherosclerosis.^232^ Hyperglycaemia also promotes atherosclerosis.^233^ The chronic inflammatory state associated with T2D is also thought to encourage plaque growth and formation.^232^ The impaired insulin secretion and action induce pathology in the microvasculature, as nitric oxide (NO) production is dependent upon insulin signalling.^234^ Chronic NO-deficiency in T2D results in a hyper-constricted state of the microvasculature; therefore, the delivery of oxygen and nutrients to tissues is impaired, contributing to diabetic neuropathies and the poor exercise tolerance observed in patients, as well as elevated blood pressure.^232^,^235^,^236^ Diabetic autonomic neuropathy (DAN) also contributes to dysregulated autoregulation of blood flow in the vasculature.^237^ Also, diabetic patients exhibit capillary basement membrane thickening due to chronic hyperglycemia, further impairing the exchange between the circulation and tissues.^232^ The excess nutrient levels in the circulation in T2D are also thought to damage cells throughout the cardiovascular system due to excessive ROS production caused by increased metabolism, as a result of increased mitochondrial nutrient supply.^232^,^238^ The cardiovascular system in T2D is also in a hypercoagulable state and patients are at increased risk of thrombosis as a result.^232^ T2D patients are also at increased risk of heart failure due to diabetic cardiomyopathy, where myocardial disease manifests in the absence of any other known CVD, likely due to hyperglycemia, microvascular damage and autonomic neuropathy associated with diabetes.^232^,^239^

**Figure 7:**
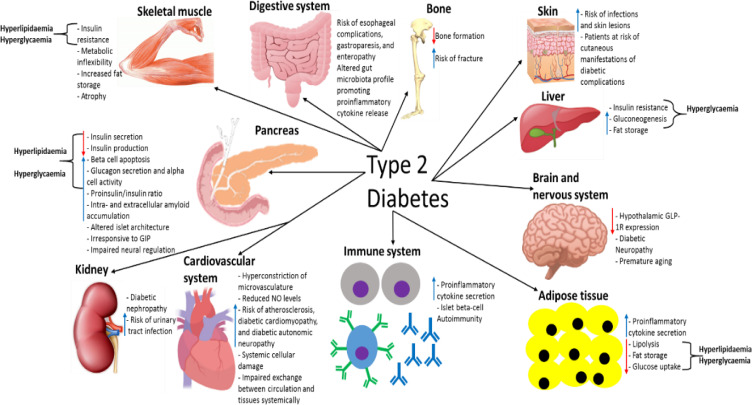
A summary of the pathological effects that T2D has on different organs and systems throughout the body, some of which directly contribute to the disease-associated dyslipidaemia/hyperglycaemia and subsequent clinical symptoms. This figure and information in its legend are with data adapted from these studies.^94^,^105^,^106^,^111^,^192–195^,^206^,^214^,^219–222^,^225^,^228^,^232^,^241^,^326–328^,^331^

Diabetic nephropathy (DN) is the most common cause of end-stage renal disease globally, with 10–20% of T2D patients developing this disease.^195^ Kidney pathology can result due to nerve and blood vessel damage in this organ caused by the diabetic phenotype.^195^,^228^,^232^ The diabetic phenotype also promotes DN by inducing pathology in kidney cells directly involved with glomerular filtration, as, for example, hyperglycemia promotes fibrosis in the kidney via inducing activation of specific intracellular pathways – the mechanisms by which T2D induces DN are reviewed in detail elsewhere.^195^,^240^ In addition, urinary tract infections are more common in diabetic patients, which is at least partly thought to be due to increased glucose levels in the urine.^241^ Figure 7 summarises the pathological effects that T2D has on different organs and systems throughout the body.

## Pharmacological Treatments and Lifestyle Changes for T2D

After the T2D diagnosis, initial treatments usually involve altering diet and lifestyle, as well as increasing physical activity in combination with metformin.^3^ Metformin decreases hepatic glycogenolysis, decreases peripheral tissue insulin resistance, increases GLP-1 postprandial secretion (which augments insulin secretion), and delays digestion.^8^ Exercise and weight loss also reduce insulin resistance and reduce the risk of T2D associated post-diagnosis complications.^242^,^243^ If this treatment regime is ineffective at promoting normoglycaemia then sulfonylureas and meglitinides are usually the next treatments tried which act as insulin secretagogues.^3^ Common side effects of insulin secretagogues include weight gain and hypoglycaemia, and these drugs are only usually effective in the short term.^8^ Insulin therapy, in the form of multiple daily subcutaneous injections, is effective at lowering plasma glucose levels and is usually required in the majority of patients when the aforementioned treatment regimens fail, but there are several side effects, such as weight gain, hypoglycaemia and increased risk of colorectal cancer.^3^,^244^ It has been estimated that 50% of patients require insulin therapy within 10 years of T2D diagnosis.^245^ Recently, rodent and human studies have found that orally administered insulin could be a viable option to treat T2D. This is due to the advent of novel delivery systems that allow oral insulin to resist degradation in the GIT, be transported into circulation, and then act when desired depending on plasma glucose concentration, which is desirable given the complications associated with multiple daily invasive insulin injections.^246–249^ Modulating GLP-1R activity is the most recent therapy used to treat T2D.^3^ The first therapy is based on the use of DPP-IV inhibitors to delay GLP-1 breakdown from its active to inactive form.^250^ DPP-IV inhibitors increase GLP-1 levels by 2–3-fold over 24 hours and have been shown to significantly decrease plasma glucose levels.^3^ Long-term administration of these drugs will likely have adverse effects as this enzyme is expressed in many tissues and has a variety of functions, but no important side effects have been seen after over 10 years of use.^3^ The efficacy of GLP-1 analogues is generally greater than DPP-IV inhibitors, due to the supraphysiological concentrations of GLP-1 achieved after administration of the former.^251^ Hence, the preferred therapy for modulating GLP-1R activity involves the administration of GLP-1 analogues (exenatide and liraglutide, lixisenatide, dulaglutide, albiglutide and semaglutide) to T2D patients, which improve glycaemic control by augmenting insulin secretion and dampening glucagon secretion, as well as delaying gastric emptying.^252^,^253^

**Figure 8:**
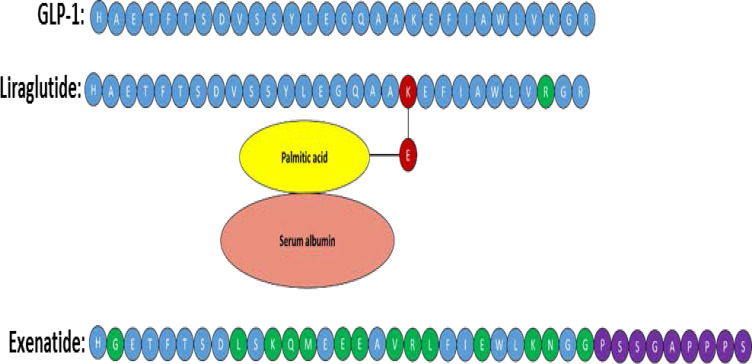
The amino acid sequence of GLP-1, liraglutide and exenatide. Amino acids highlighted green in exenatide indicate altered amino acids from the GLP-1 sequence. The purple amino acids in exenatide indicate amino acids that have been added. The amino acid highlighted red in liraglutide is modified by the addition of palmitic acid enabling binding to albumin which results in an increased half-life of liraglutide in circulation. This figure is with data adapted from Lorenz et al.^332^

Exenatide and liraglutide mimic the endogenous GLP-1 activity by binding to GLP-1R on various tissues, but these analogues are resistant to degradation.^252^ GLP-1 has a half-life of 1–2 minutes, whereas exenatide has a half-life of 3.4–4 hours and liraglutide’s half-life is 11–13 hours.^3^ Hence, these GLP-1 analogues vastly prolong the GLP-1 response promoting normoglycaemia in T2D patients during fasting and after nutrient ingestion. An advantage of using GLP-1R agonists (and DPP-IV inhibitors) is that they only mediate their insulinotropic effects when glucose levels are elevated, meaning that the risk of hypoglycaemia is minimal, and these therapies have the lowest reported rates of hypoglycaemia for T2D treatments.^254^ Liraglutide and exenatide (synthetic exendin-4) have 97 and 52%, respectively, homology with GLP-1.^255^,^256^ GLP-1R agonists used in T2D treatment are either derivatives of native GLP-1 (liraglutide, albiglutide, semaglutide and dulaglutide), which have been modified to be resistant to DPP-IV inactivation or derivatives of exendin-4 (exenatide, lixisenatide and exenatide-LR).^257^,^258^ Exendin-4 was originally isolated from the saliva of the Gila monster lizard and is resistant to the action of DPP-IV.^257^,^259^ Key pharmacological and clinical features of clinically available GLP-1R agonists are presented in Table 3 and Figure 8 compares the amino acid sequence of native GLP-1 and the GLP-1 analogues liraglutide and exenatide.

**Table 3:** Current Glucagon-Like Peptide 1 Receptor (GLP-1R) Agonists Used in Type 2 Diabetes Therapy

GLP-1R Agonist Generic Name (Trade Name)	Dosing	Half-Life	Administration Required Before Meals?
**Short-acting**			
Exenatide (Byetta)	Twice daily	2.4 hours	Yes
Lixisenatide (Lyxumia)	Once-daily	4 hours	Yes
**Intermediate-acting**			
Liraglutide (Victoza)	Once-daily	12 hours	No
**Long-acting**			
Exenatide-LAR (Bydureon)	Once weekly	96 hours	No
Albiglutide (Tanzeum)*	Once weekly	6–8 days	No
Dulaglutide (Trulicity)	Once weekly	90 hours	No
Semaglutide (Ozempic)	Once weekly	165–184 hours	No

**Notes:** Adapted with permission from Reed J, Bain S, Kanamarlapudi V. Recent advances in understandingthe role of glucagon-like peptide 1. *F1000Research*. 2020;9:239.^257^ *This product was globally withdrawn in July 2018 for commercial reasons.

These GLP-1 analogues are effective insulinotropic agents and successfully suppress glucagon secretion in a glucose-dependent manner.^260–261^ GLP-1 analogues also induce weight loss, which is associated with improved T2D prognosis.^252^,^262^,^263^ GLP-1R activity is thought to mediate weight loss by induction of satiety.^3^,^252^ Interestingly, it has been shown that chronic liraglutide treatment in diabetic mice prevents loss of pancreatic beta-cell mass, increases proliferation of the beta-cells and decreases apoptosis after alloxan injection.^264^ Inhibition of the beta-cell apoptosis in isolated human pancreatic cells was also reported after liraglutide administration, and the beta-cell proliferation rate was increased up to three-fold after incubation for 24 h.^265^ In streptozocin-induced type 1 diabetic rats and isolated human pancreatic ducts, exendin-4 treatment has also been shown to increase the number of insulin producing cells.^266^ Side effects of GLP-1 analogue administration are dyspepsia and nausea, but the effects usually subside after continuous administration.^3^ Additionally, it was implicated that GLP-1 analogue therapy increased the risk of acute pancreatitis, but subsequent studies using larger cohorts did not confirm this finding.^8^,^267^ However, there is evidence that GLP-1 analogues increase the risk of medullary thyroid cancer, which is a cause for concern.^268^

The development of GIP analogues for T2D treatment was initially not a desirable field of research due to GIP action being thought to be lost in T2D and GIPs ability to augment glucagon secretion.^3^,^261^ However, unexpectedly, preclinical data from a study in 2013 found that GLP-1/GIP dual agonist treatment induce better glycaemic control and body weight reduction in diet-induced-obesity mice compared to liraglutide only treated controls.^269^ Co-agonism of GLP-1R and GR was also shown to reduce body weight synergistically in rodent obesity models. These co-agonists also improved glycaemic control in humans.^269^ Additionally, a recent study found that co-agonism of GLP-1R and GR was reported to ameliorate diet-induced dyslipidemia and atherosclerosis by increasing energy expenditure, reducing liver inflammation and regulating bile homeostasis in rodents.^270^

A recent study found that exenatide increased adiponectin secretion from adipose tissue in both in vivo and in vitro studies.^271^ Adiponectin is a peripheral tissue insulin sensitizer, and its levels are known to decrease in obese and T2D patients.^272^ This study demonstrated that modulating GLP-1R activity has the potential to improve both insulin secretion and action in T2D and that GLP-1 analogues may also be able to induce weight loss by augmenting adiponectin secretion, as well as inducing satiety. GLP-1 analogues are as effective as second-line therapy in improving glycaemic control in patients with T2D.^273^ Reductions in glycated haemoglobin from baseline with GLP-1 analogues tend to be greater than or comparable to insulin therapy.^262^,^274^ GLP-1 analogues are also consistently more effective at inducing weight loss than most oral glucose-lowering drugs and insulin.^262^,^263^ GLP-1 analogues were also shown to confer lower hypoglycemia risk versus insulin or sulfonylureas.^262^ Additionally, GLP-1 analogues also appear to decrease the risk of cardiovascular pathology in patients, according to some studies, and preliminary data suggest they do so to a greater degree than other oral glucose-lowering drugs.^262^,^275–277^ However, two GLP-1 analogues tested in two studies demonstrated no advantage in influencing cardiovascular outcome.^278^,^279^ Recent systematic reviews and meta-analyses found that GLP-1R agonists reduced major adverse cardiovascular events in T2D patients^280–282^ and also had beneficial effects on kidney outcome.^283^ The reported varying impacts of different GLP-1R agonists on blood glucose levels, weight loss, cardiovascular outcomes and adverse effects should play an integral role when determining which agonist is best to administer to a patient depending on their medical requirements.^260^

### Small Molecule Agonists

**Figure 9:**
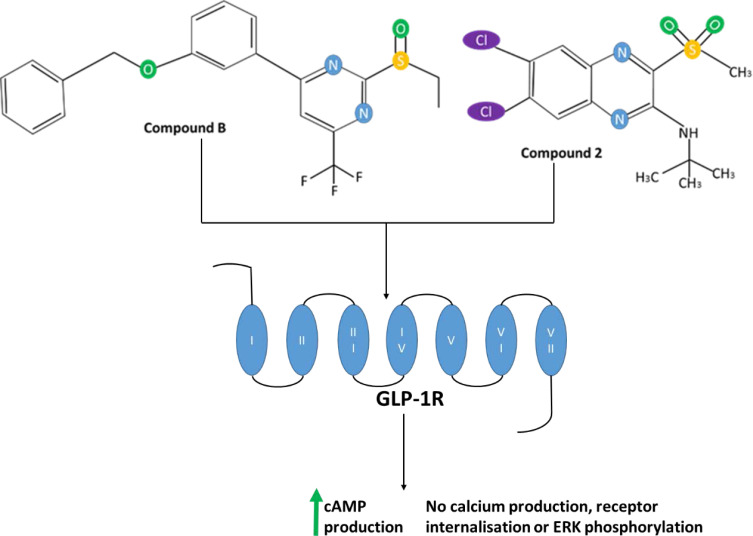
The chemical structure of the small molecule allosteric agonists compound 2 and compound B of GLP-1R, which shown schematically with numbered transmembrane domains. This figure is adapted with data from these studies.^3^,^14^

Studies have demonstrated that GLP-1R has allosteric agonist binding sites and that these sites are distinct from the orthosteric agonist (GLP-1) binding site.^3^ The first allosteric agonist identified for GLP-1R was compound 1, which had a low affinity and a low potency for GLP-1R.^3^ Compound 2 was then produced, which has been shown to be a more potent agonist, and this molecule also increases the affinity of GLP-1R for GLP-1.^284^ Additionally, compound 2 binding was not inhibited by the exendin (9–39) antagonist (truncated exenatide), demonstrating that this compound binding site on GLP-1R is distinct from the orthosteric GLP-1 binding site.^3^,^284^ However, compound 2 does not stimulate insulin secretion to the same extent as GLP-1, liraglutide or exenatide in vivo, and combining liraglutide or exenatide with compound 2 did not improve insulin secretion in mice.^285^ Compounds A and B have also been shown to demonstrate ago-allosteric properties and induce cyclic adenosine monophosphate (cAMP) signalling, as well as increased insulin secretion in rat islets and animal studies.^3^,^14^ One study showed that compound B almost normalised insulin secretion in human islets isolated from a T2D donor.^286^ Compounds 2 and B bind to a distinct site from the orthosteric binding site, as GLP-1R antagonists exendin (9–39) and JANT4, as well as the V36A mutation in GLP-1R, did not inhibit cAMP production upon compound 2 or B administration.^14^ Compounds 2 and B induced cAMP production in human GLP-1R expressing cells but caused no intracellular Ca^2+^ accumulation, ERK phosphorylation or receptor internalisation.^14^ Prevention of receptor internalisation may allow for continuous downstream signalling of the receptor, which could, in theory, raise insulin levels in T2D patients – this is an area of ongoing research.^252^ The K334A mutation (which affects the Gαs coupling) of GLP-1R inhibited both GLP-1 and compounds 2 and B induced cAMP production, which demonstrates that GLP-1 and both compounds 2 and B induce similar conformational changes needed for the Gαs activation.^14^ A recent study showed that compounds 2 and B bind and covalently modify residue 347 in ICL3 of GLP-1R.^287^ Residues 328, 351 and 335 of GLP-1R were recently demonstrated to be important for compound 2 potency, as mutagenesis of these residues reduced compound 2 activity.^288^ In this same study, enhanced compound 2 potency was demonstrated by GLP-1R when its residue 332 was mutated to tryptophan, which is in contrast to the effect observed with this mutation on the potency of allosteric antagonists. It was postulated that enhanced compound 2 potency was caused by increased hydrophobic interactions between the receptor and the agonist with this mutation. Figure 9 shows the chemical structures of compounds 2 and B.

### GLP-1R, GIPR and GR Tri-Agonist

In 2015, a triple agonist for GLP-1R, GIPR and GR was developed and tested on diet-induced obese (DIO) mice.^289^ The tri-agonist lowered body weight in these mice to a greater extent than the GLP-1R/GIPR coagonist after 20 days of treatment. Both the coagonist and tri-agonist did not induce hypoglycaemia and were equally effective at reducing blood glucose levels and improving glucose tolerance, demonstrating that surprisingly chronic GR agonism does not counteract the anti-hyperglycemic effects of GLP-1R and GIPR activity. Interestingly, tri-agonist treated mice had lower plasma insulin levels than the coagonist-treated mice, indicating improved insulin sensitivity. In theory, chronic GR activation should raise plasma glucose levels and insulin secretion would then need to be concomitantly increased to maintain normoglycaemia during tri-agonist treatment, but this was not observed in this study. Given that there was no difference in food intake between wild-type mice treated with the dual-incretin GLP-1/GIP coagonist and those treated with the tri-agonist, the greater difference in weight loss was attributed to significantly enhanced ATP expenditure in tri-agonist-treated DIO mice. The capacity of the tri-agonist to prevent the development of spontaneous diabetes compared with the dual-incretin GLP-1/GIP coagonist was tested in mouse models of T2D. Tri-agonist treatment prevented the excessive weight gain to a greater extent than the coagonist, and this difference was not due to cumulative food intake. Additionally, the tri-agonist protected these mice from fasting hyperglycaemia to a better degree than the coagonist. Interestingly, the tri-agonist also significantly reduced pancreatic alpha-cell infiltration into the core of pancreatic islets, helping to preserve the islet architecture observed in healthy pancreatic islets. The tri-agonist was also demonstrated to delay T2D progression in rodent models of spontaneous diabetes, as glycaemic improvements were maintained in Zucker diabetic fatty rats 3 weeks after treatment cessation, even though they had gained body weight and were comparable in mass to vehicle-treated controls. Given that weight and food intake were not altered in lean mice even after chronic treatment with the tri-agonist, this demonstrates that the effects of the tri-agonist do not manifest in the absence of excessive nutrient storage. Another recent study,^290^ which investigated the effects of other GLP-1, GIP and GR tri-agonist in rodents, found that this agonist ameliorated diabetic nephropathy-related disorders, as well as reduced body weight, food intake and hyperglycaemia. This suggests that these tri-agonists have the therapeutic potential to alleviate post-diagnosis complications in patients.

## Surgical Options for T2D: Bariatric Surgery

Bariatric surgery includes a variety of procedures performed on the GIT in people who are severely obese (BMI ≥40) or in individuals with a BMI of ≥35 that have a condition that can be improved by losing weight such as T2D or high blood pressure.^291^ Weight loss is achieved by reducing the size of the stomach with a gastric band or by removing a portion of the stomach (sleeve gastrectomy or biliopancreatic diversion with duodenal switch), and resecting and re-routing the small intestine to a small stomach pouch (gastric bypass surgery) is another surgical option to promote weight loss.^292^ Reduction in the size of the stomach results in patients experiencing satiety with less food and the bypass means that fewer nutrients will be ingested as part of the intestine will no longer be involved in digestion.^291^,^292^ This surgery is only used when other options have not been effective for weight reduction, such as diet and lifestyle changes.^13^,^291^ The obesity epidemic in recent decades has been parallelled by an increase in bariatric surgery.^292^ Longitudinal studies have found that this surgery significantly reduces body weight over time, improves glycaemic control and prognosis in T2D patients, and reduces mortality and risk of obesity-related co-morbidities, such as cardiovascular and respiratory pathology.^291^ Data collected globally revealed that the number of patients undergoing bariatric surgery rose from 40,000 in 1998 to 146,300 in 2003.^293^

Bariatric surgery is known to be able to induce T2D remission, and one study found that 53% of patients were in complete T2D remission three years post-surgery and another 10% were in partial remission according to the American Diabetes Association criteria, although remission rates vary between the studies, with one study reporting that 34% of patients were in complete remission 23 months post-surgery.^294^ The consensus in the literature is that complete remission rates decrease with time, as demonstrated in one study where 43% of patients had complete remission after 2 years post-surgery, which dropped to 24% after 5 years.^294^,^295^ Recent findings suggest that a persistent impairment in insulin secretion is a crucial factor in failure to sustain T2D remission after bariatric surgery.^295–297^ The improvements observed in post-surgery were originally postulated to be induced by the subsequent post-surgery weight loss, but it has become apparent that reversal of insulin resistance is induced before any significant weight loss occurs.^298^ Hence, it is currently unclear how T2D remission is achieved via bariatric surgery, but it is not just due to the weight loss associated with this surgery. In both T2D obese and T2D-free obese patients, bariatric surgery increases postprandial GLP-1 levels (from up to 3-fold pre-surgery and nearly 8-fold vs baseline after gastric bypass).^297^ Interestingly, gastric bypass and sleeve gastrectomy have been reported to induce similar improvements in glycaemic control, despite inferior GLP-1 levels and BMI decreased in patients that had undergone sleeve gastrectomy.^296^

A recent study reported that bariatric surgery improves disposition index (DI), a score determined by insulin sensitivity times the amount of insulin secreted in response to blood glucose levels, in diabetes-free subjects such that after 2 years post-surgery, it almost matches general population values.^297^ However, insulin secretion in response to intravenously administered glucose declines in these individuals, implying that the improved DI is a result of the enhanced post-surgery GLP-1 plasma levels. In the T2D subjects of the same study (91% remission rate at 2 years post-surgery), both disposition indices and insulin secretion in response to intravenously administered glucose improve 2 years post-surgery but are still notably lower than population norms. Fasting plasma glucose levels were reported to revert to normal levels 2 years post-surgery in over 90% of T2D subjects (from 7.30 to 5.24 mmol), and become comparable to non-diabetic subjects (5.00 mmol). Although average peak glucose levels remained similar in the diabetic group 2 years after surgery (from 9.96 to 9.79 mmol/L), peak postprandial glucose levels shifted to an earlier time point and rapidly declined to within the normoglycaemic range, meaning that the time the individual was subjected to hyperglycaemia was greatly reduced and for the majority of 4 hours following nutrient ingestion their plasma glucose levels were in the healthy range. Surprisingly, average peak glucose levels increased in non-diabetic subjects after surgery, but these individuals similarly had peak levels at an earlier time point, with a subsequent more rapid decline in plasma glucose levels. Despite this postprandial hyperglycaemia, the overall reductions in fasting and postprandial glucose exposure following bariatric surgery in nondiabetic individuals are thought to be beneficial due to the reported reduced cardiovascular complications associated with post-surgery groups compared with non-surgical control groups.^297^ Average peak insulin levels were notably lower in diabetic subjects (771.4 pmol/L) compared to non-diabetic (1221 pmol/L) 2 years post-surgery, although average fasting insulin levels were higher in diabetic subjects (41.5 vs 36.9 pmol/L). In both groups, average peak insulin levels improved 2 years post-surgery: pre-surgery peak insulin levels were 491.9 and 7778.3 pmol/L in the diabetic and non-diabetic groups, respectively. Interestingly, although the average proinsulin: insulin ratio marginally improved 2 years after surgery in diabetic subjects (from 2.2 to 1.8), it remained notably higher than the value observed in the non-diabetic control group (1.1), demonstrating that bariatric surgery does not have much impact on defective insulin processing by islet beta-cells. Interestingly, average peak postprandial glucagon levels increased in both diabetic (from 102.2 to 112.3ng/L) and non-diabetic (from 99.2 to 109.9 ng/L) groups after surgery, implying a beneficial role of this hormone in promoting post-surgical weight loss, and the opposite was the case for fasting glucagon levels.

Plasma GLP-1 and GIP responses, as well as insulin levels, were augmented in a pattern that mirrored the accelerated postprandial glucose appearance and disappearance following surgery in both diabetic and nondiabetic groups. Despite the notable benefits and reported metabolic improvements, this surgery is expensive and not always successful in inducing weight loss. Further, there are numerous post-surgery complications, such as vomiting and dumping syndrome, iron and B12 deficiency, and secondary hyperparathyroidism.^13^ The majority of patients develop vomiting and dumping syndrome post-surgery but this usually subsides within a year due to patients' GIT and feeding behaviours adjusting, and medication is available to treat this syndrome if necessary.^13^ After one year post-surgery, ~30% of patients need B12 supplementation to maintain normal plasma levels of B12.^299^ The prevalence of B12 deficiency increases in the post-surgery population yearly, it has even been reported to be as high as 70% in one study in the long term.^300^ B12 supplementation usually treats this post-surgery complication, but a minority of patients require monthly intramuscular B12 injections.^13^ Iron deficiency is usually only seen in menstruating women after one year of post-surgery, and this complication can be overcome by intravenous iron infusion several times a year.^13^,^301^ During weight loss after surgery, many patients develop gallstones, and patients that develop gallstone-related symptoms undergo surgery to remove the gall bladder.^13^ Secondary hyperparathyroidism is characterised by elevated levels of parathyroid hormone caused by excessive activity and hyperplasia of the parathyroid glands.^302^ This hormone has catabolic effects on bone tissue and increases vitamin D synthesis, which raises the plasma calcium levels.^303^ Bariatric surgery patients often develop secondary hyperparathyroidism due to malabsorption of calcium from the GIT as a result of the surgery.^13^ Vitamin D and calcium supplementation are effective treatments for this post-surgical complication.^304^ It has been estimated that ~29% of patients that undergo bariatric surgery develop secondary hyperparathyroidism.^13^ Thus, it is desirable for a treatment to be developed to mimic the effects of bariatric surgery but without the aforementioned post-surgery complications.

## Future Perspectives

Studies have found evidence that GLP-1 exerts multiple beneficial extrapancreatic actions on both tissues positive and negative for GLP-1R expression,^252^ and clinical trials have found that some GLP-1 mimetic-based therapies alleviate cardiovascular pathology associated with T2D.^305^ This implies that the GLP-1R agonists may be able to alleviate the systemic pathology that is associated with T2D,^306–308^ and not just be able to augment insulin secretion from islet beta-cells of T2D patients.

In addition to augmenting insulin secretion from islet beta-cells, both in vivo and in vitro studies have produced evidence that GLP-1 action improves islet beta-cell survival and proliferation,^252^,^259^ which is also a relevant area of research as it is well established that T2D pathology is associated with an increased rate of islet beta-cell apoptosis.^309^ Given the reduced population of these cells in patients,^310^ enhanced proliferation would, in theory, be desirable to increase plasma insulin levels in patients to promote normoglycaemia and normolipidaemia. Therefore, comparing how GLP-1R orthosteric and allosteric agonists influence islet beta-cell survival is a desirable future area of research. Despite controversial evidence that islet alpha-cells express GLP-1R, there is substantial evidence to suggest that GLP-1 action suppresses glucagon secretion, which is also important for alleviating T2D pathology, as T2D is now considered to be a bi-hormonal disorder.^142^ Therefore, determining how GLP-1R orthosteric and allosteric agonists modulate islet alpha-cell activity is another area of future research. Finally, to obtain further insight into how orthosteric and allosteric GLP-1R agonists can modulate islet beta-cells, it will be necessary to determine how these agonists influence other cells in the pancreatic islets, such as alpha-cells and determine how these cells, in turn, may subsequently modulate beta-cell activity via paracrine signalling.

GLP-1R orthosteric agonism has been shown to be an effective treatment for T2D, and the therapeutic potential of GLP-1R allosteric agonism to provide more efficacious treatment is an ongoing area of research. Although not discussed here, modulating the activity of other cell receptors via allosteric agonism could also yield more efficacious therapeutic options for T2D. Modulating the activity of multiple receptors (including GLP-1R) simultaneously for hormones involved in metabolic homeostasis using a unimolecular agonist appears to be a promising new therapeutic option for future T2D treatment, and is an area of ongoing research.^289^,^290^ Extrapancreatic pathology associated with T2D is discussed in this review to highlight that this disease has systemic consequences on the body, and ideally, treatments should aim to alleviate this systemic pathology via direct mechanisms as well as by improving insulin secretion/action; given that GLP-1 targets multiple organs, GLP-1-based therapies could achieve this.^252^ A recent longitudinal study demonstrated the potential of GLP-1R agonism to prevent T2D manifestation in obese individuals and individuals with a BMI of >27 that had hypertension or dyslipidaemia: after 160 weeks, the liraglutide treated group (n=1472) had a smaller percentage of the population diagnosed with T2D (2% vs 6%) than the placebo-treated control group (n=738).^311^ Additionally, time for T2D onset during the 160-week study was found to be 2.7x longer in the liraglutide treated group than in the placebo control group. The results of this study have important future implications, as GLP-1R agonism may not only be an effective treatment for T2D but also prevent or delay the disease manifestation in at-risk individuals. A recent study also found that a new GLP-1R agonist, peptide 8, had a greater insulinotropic effect (at 30nM) than GLP-1 on rat islets, and this agonist was also shown to have effects similar to exendin-4 in mice during an OGTT.^312^ However, peptide 8 had a significant glucose-lowering effect even when it was administered 30 min before the OGTT, in contrast to exendin-4, due to its extended duration of action. The clinical potential of peptide 8 is another area of future research, given its enhanced insulinotropic activity over GLP-1 and other GLP-1R orthosteric agonists tested in this study. Given the different structures of GLP-1R agonists, it is plausible that how they bind to GLP-1R differs, which subsequently produces differential activation of the receptor, explaining the observations from studies.^288^,^312^ Further understanding mechanistically how GLP-1R can be activated differently by different orthosteric and allosteric agonists may have clinical relevance in the future. Given the conflicting findings observed between human and animal studies investigating the activity of hormones involved in regulating metabolic homeostasis (especially GLP-1), it is important to note that observations from animal studies may have limited clinical implications for developing new T2D therapies for humans.^257^,^305^

Interestingly, over recent years, it has become increasingly recognised that the aetiology of T2D encompasses autoimmune activity, similar to T1D.^313^ Strikingly, studies have demonstrated that islet-reactive T-cells can be found in phenotypic human T2D patients, and many studies have found that a subset of T2D patients test positive for islet beta-cell autoantibodies - these T-cells and antibodies are usually associated with T1D pathogenesis.^313^ One study found that 12% of T2D patients screened for islet autoantibodies were positive for at least one islet autoantibody.^314^ Staggeringly, islet reactive T-cells were found in 72% of phenotypic T2D patients that participated in one study, which was an unexpected finding, especially as great care was taken to select patients that had minimal chance of being misdiagnosed with T2D and have T1D - all participants were obese or had an increased waist-to-hip ratio, had no history of ketoacidosis, and had not received insulin treatment.^315^ Convincing evidence of autoimmunity being involved in T2D pathogenesis was also provided by a study that induced B lymphocyte deficiency in New Zealand obese mice (an obese polygenic mouse model of obesity and T2D), as B lymphocyte deficient mice were found to not develop T2D - this implies that B cells may be vital for T2D pathogenesis.^316^ Further evidence of B lymphocyte involvement in T2D pathogenesis has been provided by the observation that the B cells of T2D patients fail to secrete IL-10 (an important negative immunoregulatory molecule) after stimulation.^317^ The notion that T1D and T2D may not have such distinct aetiologies as previously thought is certainly an area for future research and is of great interest. If it becomes elucidated that T2D does have an autoimmune aetiology then it can be classified as a less severe variant of T1D, as T2D patients do not exhibit the absolute insulin deficiency seen in T1D. Additionally, if both T1D and T2D have similar autoimmune aetiologies, understanding why T2D patients do not have the T1D clinical phenotype is an important area of future research.

A recent study found by using live-cell imaging that the ability of insulin vesicles to successfully dock onto the plasma membrane correlates with insulin secretion from human islet beta-cells and this was shown to be reduced in T2D.^318^ This study found that defective insulin secretion in T2D islet beta-cells was caused by a drastically decreased rate of successful docking events (docking events were found to be 5-fold more frequent in non-diabetic beta-cells compared to diabetic beta-cells), rather than reduced delivery of insulin vesicles to the plasma membrane, which was found to be similar between non-diabetic and diabetic beta-cells. Expression analysis found that the expression of proteins involved in granule docking was downregulated in T2D. Hence, this suggests in T2D that defective molecular attachment of insulin vesicles to the release site results in inadequate plasma insulin levels to promote normoglycaemia, and that the diabetic phenotype is not as a result of intracellular insulin content and/or the number of insulin vesicles being insufficient in diabetic islet beta-cells. Improving islet beta-cell granule docking, therefore, represents an attractive and new therapeutic target for the development of antidiabetic therapies.

A recent systematic review and meta-analysis reported that using hematopoietic stem cells and mesenchymal stem cells derived from bone marrow, placenta or umbilical cord tissue to treat T2D is generally safe. The stem cell therapy also improves one or more of the following for T2D patients: levels of C-peptide, HbA1c, quality of life score, and insulin requirements.^319–321^ However, there have been inconsistencies with these findings generated in other studies and one study reported severe infections in T1D patients after the stem cell therapy.^319^,^322–324^ Additionally, the mechanism by which these stem cells exert any positive effects is debated. Also, the studies that were used to generate these findings had several design flaws with regard to deducing accurate conclusions and the total sample size was not large enough.^319^ The encouraging preliminary results from these studies warrant validation in larger, randomized, double-blind studies, as well as longer follow-up periods to establish the possibility of stem cell-based therapies for future T2D treatment.

## Conclusion

A better understanding of metabolic homeostasis in healthy individuals and the altered metabolic phenotype in T2D will likely lead to the development of better treatments for T2D. The role of the nervous system, genetics, hormones involved in metabolic homeostasis (such as insulin, glucagon, GLP-1 and GIP), glucolipotoxicity diets and feeding behaviours, sedentary lifestyles, altered islet architecture, the immune system, altered islet-cell behaviour, UCP2, altered extrapancreatic behaviour and risk factors (such as psychological stress) have in T2D aetiology and pathogenesis remains to be mechanistically understood. Given that T2D is a multifactorial disease involving an array of hormones, their receptors and subsequent intracellular activity, future therapeutic research needs to take into account how the action of all of these hormones interact synergistically in T2D to produce the altered metabolic phenotype, and also how treatments such as GLP-1R activation-based therapies can influence this hormonal synergism to produce a metabolic phenotype more similar to that of a healthy individual. GLP-1R agonists are an attractive target to generate more effective therapies for T2D given that they have been reported to have beneficial effects on multiple organs in the body, which are involved in disease pathology.

## Abbreviations

ADP/ATP, adenosine diphosphate/triphosphate; BMI, Body mass index; cAMP, cyclic adenosine monophosphate; CVD, Cardiovascular disease; CNS, Central nervous system; DN, Diabetic nephropathy; DIO, Diet-induced obese; DPP, Dipeptidyl peptidase; GIP, Gastric inhibitory peptide; GIPR, Gastric inhibitory peptide receptor; GIT, Gastrointestinal tract; GLP-1, Glucagon-like peptide-1 GLP-1, glucagon-like peptide-1; GLP-1R, GLP-1 receptor; GSIS, glucose-stimulated insulin secretion; GR, Glucagon receptor; HDL, High-density lipoprotein; IAPP, Islet amyloid polypeptide; IFG, Impaired fasting glucose; IGF, Insulin-like growth factor; IR, Insulin receptor; LDL, Low-density lipoprotein; NAFLD, Non-alcoholic fatty liver disease; NO, Nitric Oxide; PPG, Preproglucagon; PMF, Proton motive force; ROS, Reactive oxygen species; T1D/T2D, type 1/2 diabetes; UCP1/2, Uncoupler protein 1/2; WHO, World Health Organisation.
