# A Review of Current Trends with Type 2 Diabetes Epidemiology, Aetiology, Pathogenesis, Treatments and Future Perspectives [Corrigendum]

**DOI:** 10.2147/DMSO.S633326

**Published:** 2026-07-09

Reed J, Bain S, Kanamarlapudi V. *Diabetes Metab Syndr Obes*. 2021;14:3567–3602. https://doi.org/10.2147/DMSO.S319895

The authors have identified incorrect references throughout the published paper.

More references should have been included in the Figure 3 legend on page 3574. The last sentence of the figure legend should read “This figure and information in its legend are with data adapted from these studies.^98,99,101,103,105,106,111,142,143,157,163,326–328^”

On page 3580, second paragraph, there are some incorrect references. Quoted text below from the review is in *italic*.

1) *Isolating islets from diabetic rodents and exposing them to euglycemic conditions for 8–12 h resulted in the return of both normal insulin secretory capacity and beta-cell function.^89^*Reference 89 here is incorrect. The correct reference should be: Alarcon C, Boland BB, Ucgizono Y, et al. Pancreatic β-Cell Adaptive Plasticity in Obesity Increases Insulin Production but Adversely Affects Secretory Function. *Diabetes*. 2015;65(2):438–450. doi: 10.2337/db15-0792

2) *Furthermore, bariatric surgery (which results in reduced food intake post-surgery) and low-calorie diets can induce T2D remission, which can be viewed as in vivo evidence that reducing demand on the beta-cell (ie, “beta-cell rest”) improves endogenous insulin secretory capacity.^98^*Reference 98 here is incorrect. The correct reference should be: Arterburn D, Bogart A, Coleman KJ, et al. Comparative Effectiveness of Bariatric Surgery versus Nonsurgical Treatment of Type 2 Diabetes among Severely Obese Adults. *Obes Res Clin Pract*. 2013;7(4):e258-268. doi: 10.1016/j.orcp.2012.08.196

3) *Administering diazoxide (which inhibits endogenous insulin secretion and therefore induces a degree of transient “beta-cell rest”) to T2D patients resulted in a 280 and 500% increase in insulin levels over baseline after stimulation tests in T2D low and high beta-cell reserve groups, respectively.^176^*Reference 176 here is incorrect. The correct reference should be: Greenwood RH, Mahler RF, Hales CN. Improvement in insulin secretion in diabetes after diazoxide. *Lancet*. 1976;307(7957):444-447. doi: 10.1016/s0140-6736(76)91473-2

4) *This study provides in vivo evidence that “beta-cell rest” can at least temporarily allow insulin secretory capacity recovery, and in vitro studies examining the effect of diazoxide on diabetic human islets produced similar findings.^177^*Reference 177 here is incorrect. The correct reference should be: Song SH, Rhodes CJ, Veldhuis JD, Butler PC. Diazoxide attenuates glucose-induced defects in first-phase insulin release and pulsatile insulin secretion in human islets. *Endocrinology*. 2003;144(8):3399-3405. doi: 10.1210/en.2003-0056

5) *Another in vivo study found comparable effects on improved beta-cell function following short-term continuous subcutaneous insulin infusion in T2D patients that were uncontrolled by conventional oral therapies.^88^*Reference 88 here is incorrect. The correct reference should be: Glaser B, Leibovich G, Nesher R, Hartling S, Binder C, Cerasi E. Improved beta-cell function after intensive insulin treatment in severe non-insulin-dependent diabetes. *Acta Endocrinol (Copenh)*. 1988;118(3):365-373. doi: 10.1530/acta.0.1180365.

On page 3584 the references in the fourth paragraph discussing preproglucagon (PPG) neurons are also incorrect. Quoted text below from the review is in *italic*.

1) *This suggests that PPG neuron-mediated circuitry has an important physiological role in promoting glycaemic control and insulin sensitivity and that neuronal activity can promote metabolic homeostasis via extrapancreatic methods.^104^*Reference 104 here is incorrect. The correct reference should be: Shi X, Chacko S, Li F, et al. Acute activation of GLP-1-expressing neurons promotes glucose homeostasis and insulin sensitivity. *Mol Metab*. 2017;6(11):1350-1359.  doi: 10.1016/j.molmet.2017.08.009

2) *Another recent study found that the activation of cholinergic preganglionic neurons in rodents via the melanocortin-4 receptor agonist lorcaserin reduced hepatic glucose production, increased glucose disposal and improved insulin sensitivity.^129^*Reference 129 here is incorrect. The correct reference should be: Burke LK, Ogunnowo-Bada E, Georgescu T, et al. Lorcaserin improves glycemic control via a melanocortin neurocircuit. *Mol Metab*. 2017;6(10):1092-1102. doi: 10.1016/j.molmet.2017.07.004

3) *Lorcaserin has also been reported to improve glycaemic control and induce weight loss in obese T2D subjects, and this drug was additionally shown to both reduce the risk of prediabetic individuals progressing to T2D and increase the likelihood of these subjects being able to revert to euglycemia.^129,231^*Reference 129 is incorrect here. The correct references should be the one from above in point 2 that needs to be added and the current reference 231 which is correct.

On page 3598, the current incorrect references 176 and 177 are not used anywhere in the review and so should not be included in the reference list.

The authors apologize for these errors.

